# Dynamic Stimulations with Bioengineered Extracellular Matrix‐Mimicking Hydrogels for Mechano Cell Reprogramming and Therapy

**DOI:** 10.1002/advs.202300670

**Published:** 2023-04-29

**Authors:** Yufeng Shou, Xin Yong Teo, Kenny Zhuoran Wu, Bingyu Bai, Arun R. K. Kumar, Jessalyn Low, Zhicheng Le, Andy Tay

**Affiliations:** ^1^ Department of Biomedical Engineering National University of Singapore Singapore 117583 Singapore; ^2^ Institute for Health Innovation and Technology National University of Singapore Singapore 117599 Singapore; ^3^ Yong Loo Lin School of Medicine National University of Singapore Singapore 117597 Singapore; ^4^ NUS Tissue Engineering Program National University of Singapore Singapore 117510 Singapore

**Keywords:** cell therapy, hydrogel, mechanical stimulation, mechanomedicine, mechanotransduction

## Abstract

Cells interact with their surrounding environment through a combination of static and dynamic mechanical signals that vary over stimulus types, intensity, space, and time. Compared to static mechanical signals such as stiffness, porosity, and topography, the current understanding on the effects of dynamic mechanical stimulations on cells remains limited, attributing to a lack of access to devices, the complexity of experimental set‐up, and data interpretation. Yet, in the pursuit of emerging translational applications (e.g., cell manufacturing for clinical treatment), it is crucial to understand how cells respond to a variety of dynamic forces that are omnipresent in vivo so that they can be exploited to enhance manufacturing and therapeutic outcomes. With a rising appreciation of the extracellular matrix (ECM) as a key regulator of biofunctions, researchers have bioengineered a suite of ECM‐mimicking hydrogels, which can be fine‐tuned with spatiotemporal mechanical cues to model complex static and dynamic mechanical profiles. This review first discusses how mechanical stimuli may impact different cellular components and the various mechanobiology pathways involved. Then, how hydrogels can be designed to incorporate static and dynamic mechanical parameters to influence cell behaviors are described. The Scopus database is also used to analyze the relative strength in evidence, ranging from strong to weak, based on number of published literatures, associated citations, and treatment significance. Additionally, the impacts of static and dynamic mechanical stimulations on clinically relevant cell types including mesenchymal stem cells, fibroblasts, and immune cells, are evaluated. The aim is to draw attention to the paucity of studies on the effects of dynamic mechanical stimuli on cells, as well as to highlight the potential of using a cocktail of various types and intensities of mechanical stimulations to influence cell fates (similar to the concept of biochemical cocktail to direct cell fate). It is envisioned that this progress report will inspire more exciting translational development of mechanoresponsive hydrogels for biomedical applications.

## Graphical abstract

Cells communicate with their surroundings through both static and dynamic mechanical signals, which vary over stimulus type, intensity, location, and duration. Current knowledge on dynamic mechanical stimulations is limited compared to static ones. For this, researchers are now developing extracellular matrix‐mimicking biofunctional hydrogels that can replicate intricate static and dynamic mechanical profiles, enhancing the understanding of cellular complex interactions.

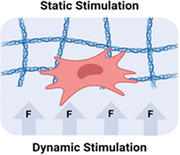

## 1 Introduction

The state and behavior of a cell are dynamic and they are a function of its intrinsic characteristics (e.g., genetic makeup, cytoskeletal contractility, and metabolic signaling pathways) and its interaction with the local environment.^[^ ^1^, ^2^ ^]^ The extracellular microenvironment consists of the extracellular matrix (ECM, neighboring cells, and a variety of biochemicals, such as hormones, growth factors, and cytokines).^[^ ^3^ ^]^ In comparison to biochemical signals, the biophysical effects of ECM in modulating cellular fates remain poorly elucidated. Notably, the biological function of ECM extends beyond inert physical support, as it can impact functional behaviors in cells such as migration, proliferation, differentiation, and apoptosis via a series of mechanical cues.^[^ ^4^, ^5^ ^]^ However, many of these findings are based on overly simplistic models that typically involve culturing cells on 2D plastic or glass surfaces that have been modified with ECM components. Such systems are unable to recapitulate the native 3D context, resulting in poor biological and clinical relevance and translatability.^[^ ^6^, ^7^ ^]^ Materials that allow in vivo cell–matrix interaction are advantageous for decoding the complex relationship between ECM and cells, and be used as a model to translate basic science findings into advances in regenerative medicine, cancer therapies for instance.^[^ ^8^, ^9^, ^10^ ^]^ In recent years, it has become evident that the ECM is more than just a passive mechanical support; it is also as an active regulator of fundamental cellular processes. While the ECM has a prominent influence over cellular processes, cells, in turn, are also continually changing ECM features by secreting ECM macromolecules or degrading the ECM network to remodel their external environment.^[^ ^10^ ^]^ This bidirectional, interdependent interaction in which cells and matrix can affect each other is referred to as ‘dynamic reciprocity’.^[^ ^11^ ^]^ Given the importance of ECM in determining cellular fates, a better understanding of cell–matrix interaction is necessary to delineate the influence of ECM on cells and vice versa, as well as to further exploit these mechanisms for tissue engineering.

A thorough understanding of the biofunctions of ECM in vivo is required for the proper design of an in vitro model to study ECM–cell interactions. The ECM serves at least five important roles. It provides i) structural support, ii) cell binding sites, iii) topographical cues, iv) porous geometries for nutrient and waste exchange, and v) tissue‐specific spatial and temporal organization to induce and maintain cellular functions.^[^ ^12^ ^]^ These biophysical properties constitute the matrix mechanics that allows cells to sense and respond through mechanotransduction.^[^ ^13^ ^]^ Mechanotransduction is typically characterized by cells binding to the surrounding matrix and applying tension across integrins, resulting in a cascade of downstream mechanosensing pathways that alter the genetic and epigenetic profile of cells and, eventually, cell fate.^[^ ^14^, ^15^ ^]^ On this note, ECM not only regulates cell behaviors, but it also undergoes dynamic structural remodeling when it interacts with resident cells.^[^ ^10^ ^]^ While understanding how ECM can direct cellular processes (e.g., embryonic development, carcinogenesis, immune response) and maintain cell functions is critical, it is extremely difficult to replicate this interaction in vitro.^[^ ^16^ ^]^

Hydrogels are a desirable material for in vitro ECM recapitulation. They are highly hydrated and porous, allowing for a large aqueous compartment as well as excellent nutrient and gas permeability, similar to in vivo tissues.^[^ ^12^ ^]^ In addition, the mechanical properties of hydrogels can be easily modified to suit a broad spectrum of tissues in the body (for example, matrix stiffness ranging from 50 Pa to 1 GPa, from the brain to bone tissue^[^ ^17^ ^]^), making them more physiologically relevant. Most importantly, many ECM proteins (e.g., laminin, collagen, fibronectin) and their associated protein derivatives (e.g., RGD, GFOGER, YIGSR) can be incorporated into the gel formulation to yield the gels properties that are similar to native ECM.^[^ ^18^, ^19^, ^20^ ^]^ To study cell–ECM mechanocommunications using hydrogels, cells are typically cultured within gels with fixed mechanical properties. Mechanical properties include stiffness, strain, porosity, topography, and cell adhesivity have been extensively studied under static settings.^[^ ^10^, ^21^, ^22^ ^]^ In addition to mimicking the dynamic structures of ECM, stimuli‐responsive hydrogels with variable mechanical properties modulated by changes in pH, temperature, light, and magnetic field are gaining popularity.^[^ ^23^, ^24^ ^]^ These stimuli‐responsive hydrogels have been shown in proof‐of‐concept studies to direct targeted cellular responses, and some have even led to appreciable outcomes for disease treatment.^[^ ^25^ ^]^

There are significant technical obstacles to replicate the native ECM in vitro. One of the major difficulties is simulating the native dynamic structural remodeling of ECM. Although some existing gel formulations, as described above, allow on‐demand self‐reconfiguration in response to external stimulations, these changes are typically one‐time only and irreversible (e.g., stiffening only).^[^ ^26^ ^]^ Furthermore, ECM is of high heterogeneity and structurally anisotropic,^[^ ^27^ ^]^ but most current hydrogel designs are mechanically identical in all dimensions, and thus unsuitable to realize spatial/temporal heterogeneity that occurs naturally in vivo. While there are hurdles, advances in material design and fabrication (e.g., additive manufacturing) have shown promise in creating in creating the next generation of hydrogels that are more structurally defined and controllable.^[^ ^28^, ^29^, ^30^ ^]^ A better understanding of the mechanotransduction process will definitely aid in the development of new material design principle (gradient hydrogel).

Here, we present a comprehensive overview of recent advances in hydrogel engineering for cell fate manipulation. First, we will discuss how mechanical stimuli can impact different cellular components and the underlying mechanobiology pathways. The principles of designing a mechanoresponsive hydrogel to modulate functions of different cell types are then described. To the best of our knowledge, this is the first comprehensive study summarizing cell mechanomodulation spanning both the fields of static and dynamic stimulations using hydrogel materials. Based on cellular differences in response to mechanostimulation (treated vs. control) and number of related published literature and citations from *Scopus* database, we categorize the rigor of evidence describing the impact of static and dynamic mechanical stimuli on clinically relevant cell types including mesenchymal stem cells, fibroblasts, epithelial cells, immune cells, and endothelial cells as ‘strong’, ‘medium’, and ‘weak’. We believe this classification will provide readers with evidence‐based recommendations on the type of mechanical stimuli to incorporate into their hydrogels for specific experimental goals. Besides, we highlight the synergistic effects of integrating multiple mechanical stimulations on different cell types to demonstrate the power of using a cocktail of different types and intensities of mechanical stimulations to influence cell fates, similar to the concept of biochemical cocktail to direct cell fate. We believe this progress report will inspire future breakthroughs in hydrogel engineering and mechanical stimuli with promising translational potential in biomedicine.

## 2 Mechanical Stimuli and Cell Behaviors

Human cells have a sense of ‘touch’, allowing them to ‘feel’ and ‘respond’ to the mechanical properties of their surroundings.^[^ ^31^ ^]^ Living cells are largely influenced by the static and dynamic mechanical characteristics of their microenvironment (e.g., ECM) and the mechanical forces that surround them (e.g., shear force from blood flow).^[^ ^32^, ^33^, ^34^ ^]^ In response to these mechanical cues, cells modify their behaviors to adapt to new microenvironment and achieve homeostasis. Mechanical stimuli have the ability to control virtually every aspects of cell behavior, including morphogenesis, proliferation, differentiation, migration, and gene/protein expression.^[^ ^32^, ^35^, ^36^ ^]^ The behaviors are cell‐type specific, which means that different types of cells could react differently even when subjected to the same stimuli type and intensity. In this regard, mechanical cues are an important informational system for regulating cell behaviors in vivo. It also implies the potential of using a cocktail of different types and intensities of mechanical stimulations to influence cell fates, similar to the concept of biochemical cocktail to direct cell fate.

### 2.1 Mechanosensation of Different Cellular Components

**Figure 1:**
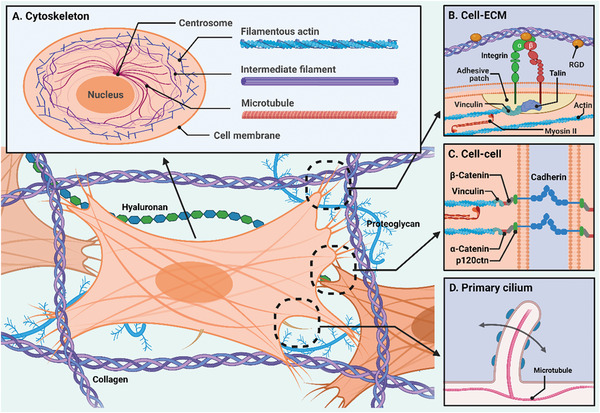
Schematic illustration of key structures of cell mechanobiological functions. A) Cytoskeleton is the dynamic network of interlinking three filamentous proteins including filamentous actin, intermediate filament, and microtubule in cytoplasm, which is the key structure for mechanical sensing and cell structural support. B) The cell–extracellular matrix interaction is transmitted to cells through integrin on focal adhesion sites. C) Cadherin on adherens junctions forms efficient intracellular adhesion and mechanical force transmission. D) Primary cilium on the cell membrane probes and collects information about the surrounding environment.

By integrating multiple cellular structures (i.e., mechanosensors) and pathways, cells can probe mechanical cues and provide specific feedback.^[^ ^37^, ^38^ ^]^ The cytoskeleton is a dynamic network of interlinking filamentous proteins (i.e., filamentous actin, intermediate filament, and microtubule) in the cytoplasm that serves as the key structure for cell mechanobiological function (**Figure** **1**A).^[^ ^39^, ^40^ ^]^ Cytoskeleton not only serves an internal scaffold as mechanical support of cell shapes and mechanical resistance to the deformation of plasma membrane, it also connects nuclear matrix to the ECM for mechanotransduction.^[^ ^37^ ^]^ As previously demonstrated, filamentous actin (F‐actin) is the primary regulator and transmitter of external mechanical stimulation to cells.^[^ ^41^ ^]^ In most eukaryotic cells, the F‐actin cytoskeleton drives cell morphogenesis changes in conjunction with the force exertion activity of myosin (an ATP‐consumed molecular motor). The process influences a diverse range of cellular processes such as cell adhesion, movement, and division by generating contractility and protrusion, thus enabling the cells to adapt to the sensed physical forces exerted on plasma membrane.^[^ ^42^, ^43^ ^]^

As the barrier that separates the interior of cells from the exterior microenvironment, the cell membrane plays a crucial role in cell mechanosensation as well. On the lipid bilayer of cellular membrane, there are several mechanosensitive microstructures, molecules, proteins, and channels. Integrin, a transmembrane heterodimer consisting of *α*‐ and *β*‐chain subunits, is the primary cell‐adhesion receptor that binds to the matrix, forming focal adhesion sites.^[^ ^15^ ^]^ It also serves as a transmembrane transmitter to the actin cytoskeleton (actin stress fiber) (Figure 1B). While one end of an integrin binds to ECM proteins for anchorage, the opposite end is usually connected to the cytoskeleton via linkage with cytoplasmic proteins like talin, filamin, vinculin, and actinin.^[^ ^44^ ^]^ These cytoplasmic proteins bind directly to actin filaments, allowing integrins and their associated proteins to mediate ECM–cytoskeleton interactions.^[^ ^45^ ^]^ When integrins bind to ECM proteins, they recruit other integrins to form a cluster which then assembles into focal adhesion complexes.^[^ ^46^ ^]^ When the cell membrane is subject to external forces, the extracellular *α*‐chain of integrin, which binds to cell‐adhesion peptide motif such as RGD sequence (i.e., tripeptide arginine–glycine–aspartate), is activated via conformational switches.^[^ ^15^ ^]^ The mechanical signals are then transmitted to the linked F‐actin cytoskeleton by the intercellular *β*‐chain subunit,^[^ ^47^ ^]^ and the cellular internal force (e.g., contractility) can then transmit to the ECM via integrin‐based focal adhesion sites.^[^ ^14^ ^]^ Mechanical cues (force transmission from the ECM) relayed by focal adhesion can directly influence cytoskeletal configuration and actin polymerization, causing gene expression and cellular responses to be perturbed.^[^ ^48^ ^]^ It should be noted that structures of focal adhesion complexes are not static because they can dynamically disassemble and reassemble in response to cytoskeleton contractility.^[^ ^49^ ^]^ In this case, the signals are sent in the reverse direction, which is intracellularly, to the focal adhesion site. Evidently, focal adhesion serves as an integrated signaling center for the transmission of mechanical, biochemical, and contextual cues, with signaling directed both inside‐out and outside‐in.

Aside from cell–ECM adhesion, cell–cell interaction (e.g., adheren junction, gap junction, etc.) is heavily involved in mechanosensation when cells are functionally clustered together and every single cell is mechanically affected by neighboring cells. For example, the establishment of cadherin‐based adherens junctions allows efficient intracellular adhesion and force transmission.^[^ ^50^, ^51^ ^]^ Furthermore, F‐actin‐binding proteins including *α*‐catenin and vinculin can modify the molecular conformation of cytoskeleton in response to transmitted force. Alternatively, F‐actin binding proteins can reorganize the cytoskeletal network by adjusting the actomyosin belts (myosin II) (Figure 1C). This may further induce force‐driven cell shape changes, collective cell migration, and tissue reorganization.^[^ ^50^, ^52^, ^53^ ^]^ Other than membrane proteins and molecules, primary cilium is another important cellular mechanosensor located on the cell membrane (generally situated at the cell apical surface).^[^ ^54^ ^]^ Primary cilium is the microtubule‐based flexible sensory ‘antennae’ that can probe and collect information about the surrounding environment (Figure 1D). For example, the primary cilium deforms in the presence of fluid flow and transmits mechanical information into the cell.^[^ ^55^ ^]^

**Figure 2:**
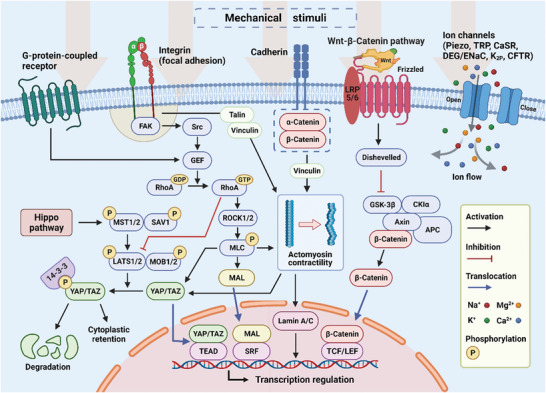
Schematic illustration of cellular mechanotransduction pathways. Matrix mechanical features and external applied forces can influence cell behaviors. The mechanical signals are transduced to the nucleus by transmembrane proteins/macromolecules, ion channels, and cytoskeleton remodeling, and then trigger alternation of gene expression to tune cell fates and activities.

Mechanosensitive ion channel is another essential force sensor present on the cell membrane. Since Hudspeth and Corey's seminal work in 1979,^[^ ^56^ ^]^ a large number of mechanically activated ion channels have been discovered. The channels consist of several mechanosensitive integral membrane proteins whose molecular conformation can be altered in response to various external mechanical forces, most notably plasma membrane tension (**Figure** **2**). These conformational changes alter the membrane permeability for specific ions, allowing them to enter the cell as mechanical signals that influence cell fates and behaviors.^[^ ^38^, ^57^ ^]^ For example, G‐protein‐coupled receptors (GPCRs) have been identified as mechanosensitive ion channels capable of detecting membrane stretching and responding to changes in conformation.^[^ ^58^ ^]^ In addition, transient receptor potential ion channels (e.g., TRPC1, TRPV4, TRPP2),^[^ ^37^, ^59^ ^]^ piezo ion channels (e.g., Piezo1 and Piezo2),^[^ ^60^ ^]^ two‐pore domain potassium (K_2P_) channels (e.g., TREK1, TREK2, TRAAK),^[^ ^61^, ^62^ ^]^ and degenerin/epithelial sodium ion (DEG/ENaC) channels^[^ ^63^ ^]^ have also been found to be mechanosensitive. Furthermore, mechanically driven ion channel activation can result in the release of signaling molecules that remodels the surrounding matrix, thereby influencing neighboring cells behavior.^[^ ^10^ ^]^

### 2.2 Cellular Mechanotransduction and Response

The cellular process by which cells integrate and convert mechanical stimuli into biochemical/molecular signals is referred to as mechanotransduction. When cells detect external forces on the cell surface, the signals can be rapidly transduced to the nucleus (through the membrane protein–cytoskeleton–nucleus axis) and trigger corresponding cellular behaviors, resulting in the formation of a robust cellular mechanoresponsive system. This force‐induced transduction also contributes to/partakes in protein conformational changes, such as the exposure of cryptic binding or signaling domains.^[^ ^64^ ^]^ Cells mediate their behaviors primarily by altering their gene expression profiles.^[^ ^65^ ^]^ Mechanosensors on the cell membrane detect mechanical cues, which are converted into biochemical signals in the cytoplasm before being relayed to the nucleus by effector proteins to regulate gene expression.

One central example of gene‐regulated mechanotransduction pathway is the Hippo pathway (Figure 2).^[^ ^66^, ^67^ ^]^ The downstream effectors in the Hippo pathway and the mechanosensitive nuclear transcription factors are mechanoactuator proteins, Yes‐associated protein and transcriptional coactivator with PDZ‐binding motif (YAP/TAZ).^[^ ^37^, ^66^ ^]^ They serve as ‘mechanical memory keepers’ by mediating gene expression and remodeling chromatin in response to external stimuli in the same way that strong or weak mechanosensitive memories do.^[^ ^68^ ^]^ When the Hippo is “switched‐on” after phosphorylation of MST1/2 by upstream effector, kinases LAT1/2 will be activated and induce phosphorylation of YAP/TAZ. Phosphorylated YAP/TAZ will be inactivated and either binds to 14‐3‐3 protein (cytoplastic retention) or degrades further. When subjected to external mechanical stimulation, signals are transmitted from membrane proteins (e.g., integrin, GPCR) to activate RhoA (transforming protein). The RhoA then inhibits LATS kinase activity (switches off the Hippo pathway) and downregulates LATS‐driven YAP/TAZ phosphorylation. This causes YAP/TAZ activation, and the proteins translocate into the nucleus (i.e., nuclear shuttling proteins), where they interact with TEAD factors to mediate gene expression.^[^ ^44^, ^67^, ^69^, ^70^, ^71^, ^72^ ^]^ As such, the location of YAP/TAZ is generally used to assess the level of mechanical signaling. For example, when cells experience low levels of mechanical stimulation (e.g., in soft ECM), most YAP/TAZ proteins are found in the cytoplasm; when cells are subjected to high levels of stimulation (e.g., in stiff ECM), many YAP/TAZ are found in the nucleus instead (i.e., nuclear localization). In addition to mechanotransduction, Hippo pathway also plays an important role in regulating cell behaviors (e.g., cell movement and growth) and is a key regulator in cancer progression, as discussed in published reviews.^[^ ^70^, ^73^, ^74^ ^]^

RhoA/ROCK pathway is another well‐researched pathway for cellular mechanotransduction (Figure 2). Under RhoA/ROCK pathway, the guanine nucleotide‐exchange factor (GEF) is activated and interacts with RhoA after the relevant membrane proteins receive external mechanical signals. In this process, GEF stimulates RhoA by catalyzing free nucleotide uptake (i.e., GDP to GTP).^[^ ^75^, ^76^ ^]^ Following that, RhoA‐GTP activates its downstream effectors, ROCK 1/2, which promotes phosphorylation of myosin light chain to induce actin stress fibers formation and actomyosin contraction. The induced intrinsic force from the cytoskeleton can directly influence transcription in the nucleus via nuclear lamina (e.g., lamin A) or activate transcription regulators (e.g., megakaryocytic acute leukaemia (MAL)) and translocate them into the nucleus.^[^ ^36^, ^77^ ^]^ SRF‐MAL, which is associated with serum response factor (SRF) in the nucleus, drives changes in gene expression and mediates cell behaviors. In addition to changes in gene expression, the cytoskeleton is altered during RhoA/ROCK pathway activation, which results in corresponding changes in cell morphology or motility. Interestingly, the transcription regulator YAP/TAZ is also stimulated in this process,^[^ ^66^ ^]^ indicating the potential synergistic effect between Hippo and RhoA/ROCK pathways. This encourages further research into the interaction of various mechanotransduction pathways to better understand how they interact in response to external mechanical stimuli. Besides, Wnt‐*β*‐catenin pathway is another important route for cellular response to external mechanical cues. *β*‐catenin is not a mere structural protein in the cadherin adhesion complex, but rather a nuclear shuttling protein that, when activated, can translocate into the cell nucleus and bind to TCF/LEF (transcription factors) to regulate gene expression (Figure 2).^[^ ^36^, ^78^, ^79^, ^80^ ^]^

In addition to cytoskeleton remodeling and gene expression alterations, changes in nuclear morphology, including shape and size, are prominent cellular responses to mechanical cues from the surrounding environment. Because the inner membrane of the nucleus is linked to the cytoskeleton, any mechanically induced cytoskeleton remodeling could lead to changes in nuclear morphology. For example, the nuclear size increases as microtubules polymerize, indicating a mechanosensitive response to the ECM stiffness.^[^ ^81^ ^]^ Recently, it was discovered that a complex known as linker of the nucleoskeleton and cytoskeleton is capable of stretching nuclear pores by transducing ECM mechanical cues to nucleus via cytoskeleton.^[^ ^82^, ^83^ ^]^ As previously stated, nucleus‐bound physical transduction pathway through cytoskeleton is much faster than the typical mechanotransduction signal pathways (i.e., chemical signals).^[^ ^84^ ^]^ As a result, the cell's initial response to mechanical stimuli is assumed to be purely mechanical, involving cytoskeleton remodeling and changes in nuclear shape.^[^ ^37^ ^]^ Another important pathway for cellular response mechanotransduction is changes in secondary messenger signaling, such as calcium ion flow (Figure 2). Readers are encouraged to refer to published review papers for more information.^[^ ^38^, ^57^ ^]^

### 2.3 Static and Dynamic Mechanical Stimuli

Mechanical stimuli, like biochemical/chemical signals, influence cellular function and growth in vivo.^[^ ^36^ ^]^ They play vital roles in the regulation of cellular physiological processes at the molecular, cellular, and systemic levels. Static mechanical stimulus is a type of mechanical factor determined primarily by the physical properties of attached substrates, matrices, or scaffolds (e.g., ECM or artificial implant materials). The stiffness of matrix is a well‐known mechanical factor that influences cell behaviors.^[^ ^85^ ^]^ Cells can sense stiffness through focal adhesion sites and transduce signals via membrane protein channels and the cytoskeleton. In the human body, a diverse range of cells and tissues reside in/on mechanical environments of varying stiffness, and the local stiffness of the matrix has a significant impact on cell behaviors.^[^ ^86^ ^]^ For example, mesenchymal stem cell differentiation to osteoblasts or chondrocytes can be regulated by substrate stiffness, which is largely mediated by mechanosensitive integrin *β*1.^[^ ^87^ ^]^ Creating an environment with appropriate stiffness has become a prerequisite for cell culture to achieve optimal cell growth and biofunctions for in vitro and in vivo work, inspired by the stiffness‐dependent attributes of the cell.^[^ ^32^ ^]^ The stiffness of artificial matrix can be easily increased by raising material mass concentration, molecular weight, and crosslinking density.^[^ ^12^, ^88^ ^]^ It is well‐known that the tumor matrix, which contains cancer‐associated fibroblasts, has a higher rigidity than normal ECM, which contributes significantly to tumor growth and progression.^[^ ^85^ ^]^ Therefore, enhanced tissue stiffness is regarded as a distinguishing feature of solid tumors.^[^ ^89^ ^]^ Furthermore, porous structure are important mechanical properties of matrices because of their strong correlation with matrix stiffness. According to recent studies, the mesh size and porosity of the matrix could influence cell proliferation and mobility.^[^ ^35^ ^]^ Lin et al. demonstrated that increasing the mesh size of hydrogel could promote cell growth.^[^ ^90^ ^]^ Additionally, substrate surface topography, morphology, and fibrous architecture are other important static mechanical features to consider in cellular mechanobiology.^[^ ^35^ ^]^

Dynamic mechanical stimulus, or externally applied force, is another important type of mechanical factor in cell mechanobiology. Cells and tissues are constantly experiencing a variety of forces within the body such as dynamic pressure produced by blood flow, skin deformation, muscle stretching, and lung expansion, all of which are essential for maintaining proper body physiology. In comparison to the mechanical characteristics of matrix or substrate, dynamic mechanical stimulation is still a relatively new field, but it is attracting greater attention in recent years. Some emerging techniques, such as microfluidic platforms and hydrogel engineering have enabled researchers to study cell behaviors under various mechanical stimulations in a simplified and accurate manner.^[^ ^34^, ^91^, ^92^ ^]^

Flow shear stress is the physical force caused by the friction of fluid flow against the cell membrane (i.e., flow‐induced shear stress). In the human body, many tissues and adherent cells are subjected to mechanical shear force in the biofluidic systems (e.g., blood and lymphatic systems).^[^ ^93^ ^]^ This mechanical stimulus has a significant impact on cell activity and adhesion. To illustrate, cells respond to shear forces by altering membrane properties (e.g., permeability), gene expression, cell behaviors, and even reorganizing the entire cell layer. In addition, abnormal flow‐induced shear stress has a significant impact on disease progression. For example, high wall shear stress from blood flow has been shown to improve the viability of human colorectal carcinoma cells due to increased intracellular signaling.^[^ ^94^ ^]^ Dynamic compression and tension forces are essential dynamic mechanical stimuli^[^ ^95^ ^]^ and they include external loading during human locomotion, and traction and adhesion forces during cell–cell/cell–ECM interactions. In recent years, a collection of studies has been conducted to investigate how tension force affects cell behavior, including YAP/TAZ nuclear localization,^[^ ^96^ ^]^ cell mobility,^[^ ^97^ ^]^ and cell adaptability.^[^ ^98^ ^]^ Similar to shear stress, abnormal tissue tension can also drive tumor aggression and progression, which is extensively reviewed elsewhere.^[^ ^99^, ^100^ ^]^

On the other hand, the effects of other in vivo mechanical stimuli, such as gravity, hydrostatic pressure, and osmotic pressure on cell behaviors cannot be overlooked. As cells in the body operate in complex mechanochemical environments, it is important to study how they respond to a combination of static and dynamic mechanical stimuli (i.e., mechanical cocktail).^[^ ^32^ ^]^ Nonetheless, the effect of dynamic mechanical stimuli on cells is currently understudied. As a result, additional research should be conducted using biomaterials such as hydrogel, which will be described in greater detail in the following section.

## 3 Current Design Principles for Mechanoresponsive Hydrogels

**Figure 3:**
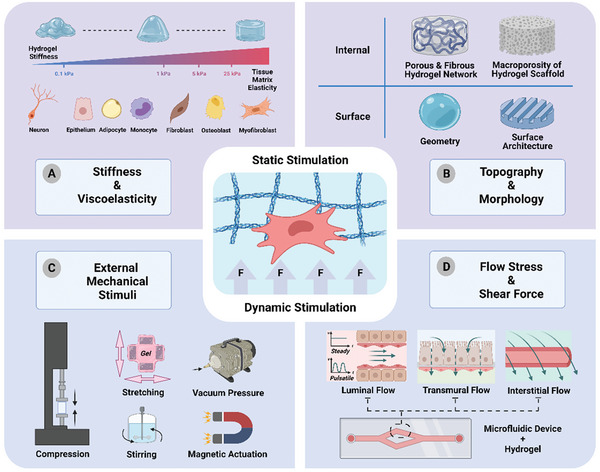
Schematic illustration of static and dynamic mechanical stimulation for hydrogel materials. A) Through modulating stiffness, hydrogels can mimic specific tissue matrix elasticity to suit different cell types.^[^ ^86^, ^113^ ^]^ B) The topography and morphology of hydrogel such as internal structures (e.g., porous and fibrous hydrogel network and macroporosity of hydrogel scaffold) and surface structures (e.g., 3D hydrogel geometry, surface architecture) influence material physical features and cell behaviors. C) Dynamic mechanical stimuli exerted on hydrogel materials by external equipment, including compression, stretching, stirring, vacuum pressure, and magnetic field. D) Microfluidic device/hydrogel‐integrated platform provides flow‐induced shear stress and mimics different in vivo flow patterns (e.g., steady or pulsatile luminal flow, transmural flow, interstitial flow).^[^ ^111^ ^]^

Hydrogels are described as 3D networks of highly crosslinked, hydrophilic polymers capable of absorbing solvents up to a few thousand heavier than their original weight.^[^ ^101^ ^]^ They can be classified based on their material origins, preparation methods, types of precursors, crosslinking methods, biodegradability, ionic charges, physical properties, and responsiveness toward external stimuli.^[^ ^102^ ^]^ Hydrogels have soft, highly hydrated properties similar to native tissue and ECM, and are capable of providing cells with an ideal environment to grow. Due to low interfacial tension, hydrogels have an exceptionally low affinity for proteins in body fluids which contributes to their high biocompatibility.^[^ ^103^ ^]^ The unique, highly porous, interconnected network structure of hydrogel is also critical for gaseous exchange, cell locomotion, as well as mass transportation of nutrients and cellular waste.^[^ ^104^ ^]^ Furthermore, the ability to tune hydrogels using a variety of methods has given researchers rigorous control over their mechanical and biochemical properties, thus bridging the gap between fabricated hydrogel scaffolds and native ECM with more realistic emulation of in vivo conditions (**Figure** **3**). To date, specific cellular microenvironments or cell behaviors can be recreated by tuning the mechanical properties of hydrogel. It is also possible to recapitulate dynamic biophysical environment on a hydrogel platform using external devices such as a digital stretcher and microfluidic pump.

### 3.1 2D Conventional Substrate versus 3D Hydrogel Matrix

Cells alone may not sustain and undergo cellular functions unless they are supported by ECM, the external environment to which cells are bound in vivo. The ECM is a 3D heterogeneous network consisting of collagens, proteoglycans, elastin, and glycoproteins that assemble to provide structural support and act as a reservoir for bioactive molecules.^[^ ^105^ ^]^ It should be pointed out that the “dimension” of matrix or substrate is also an important factor for cell growth. From a cell biology perspective, hydrogels could serve as a 3D matrix to better recapitulate in vivo microenvironment. The structural complexity and contact guidance that a 2D substrate (planar surface) can provide fair pale in comparison to a 3D environment. Cells can probe and detect differences in their surroundings in 2D or 3D, thereby transmitting corresponding mechanical and contextual cues by intracellular signaling pathways, which alter gene expression and result in distinctive cellular behaviors.^[^ ^106^ ^]^ In a conventional approach, cell culture is performed on 2D substrates such as Petri dishes and silicone substrates. This, however, does not completely replicate the in vivo milieu and frequently leads to compromised histological observations.^[^ ^107^ ^]^ The absence of 3D structural cues (e.g., porous structure) and physical supports (e.g., multidimensional force) commonly results in the formation of a disorganized layer of cells which do not differentiate and hierarchically assemble into functional tissue.^[^ ^108^ ^]^

Interestingly, the effects of mechanical stimulation also depend on the dimension of cell environment. The disparity of cellular responses between 2D and 3D microenvironments was well‐illustrated by a study conducted by Bisell and co‐workers, where human breast epithelial cells seeded in 3D basal membrane showed normal phenotypes and formed acini structures, whereas those cultured on 2D substrate exhibited tumorigenic features.^[^ ^109^ ^]^ On the other hand, it has been shown that under 2D conditions, a stiff substrate promoted mesenchymal stem cell spreading and activated cell migration,^[^ ^110^ ^]^ due to higher intracellular cytoskeleton tension, while under 3D conditions, a stiff matrix reduced cell spreading because of steric hindrance and adhesion site distribution.^[^ ^82^ ^]^ Additionally, in some cases where cells originate from a flat surface like endothelial cells, 2D culture system might more accurately reconstitute in situ condition.^[^ ^88^ ^]^ Increasingly, circumstances where 2D culture systems are being preferred are getting less popular, but they should be given due consideration depending on the scientific questions and applications.

### 3.2 Static Mechanical Stimulation of Hydrogel Materials

One big advantage of hydrogel material is that its static mechanical features including stiffness, porous microstructure, and topography, can be easily programmed to mimic the native microenvironment and to biophysically reprogram cells.

#### 3.2.1 Stiffness

Stiffness is one of the most important mechanical features of hydrogel, which is commonly defined as the material's resistance to deformation when subjected to external mechanical forces.^[^ ^111^ ^]^ In other words, stiffness is the indication of material softness or hardness. Stiffness of a material is generally quantified by elastic (or Young's) moduli. Physiologically, stiffness of ECM in tissue level varies from 0.1 kPa for brain tissue to 40 kPa in osteoid,^[^ ^112^ ^]^ and different cell types preferentially grow on tissue matrix with specific elasticity (Figure 3A).^[^ ^86^, ^113^ ^]^ The stiffness differential of ECM can be well assimilated in hydrogel fabrication, and a myriad of studies have validated the significance of hydrogel stiffness in dictating cell spreading, adhesion, proliferation, differentiation, and migration.^[^ ^114^, ^115^ ^]^ Furthermore, substrate rigidity guides cell locomotion in a phenomenon known as durotaxis, in which most adherent cells preferentially migrate up the stiffness gradient.^[^ ^116^, ^117^ ^]^ A common method used to engineer a matrix with varying stiffness is by controlling the crosslinking condition, thereby controlling the substrate flexibility by changing its crosslinking density. Three common ways to manipulate the mechanical stiffness of hydrogel are: changing comonomer/monomer composition, altering crosslinking agent concentration, and modulating the polymerization‐induced condition (e.g., pH, temperature, reaction time, light intensity for photopolymerization).^[^ ^118^ ^]^ Readers are encouraged to refer to published papers for the advantages and limitations of these approaches.^[^ ^25^, ^26^ ^]^

#### 3.2.2 Macroporosity of Hydrogel Network

Different from the inherent mesh size of hydrogel which is generally defined as the linear distance between two adjacent crosslinks and typically on the nanometer scale,^[^ ^119^, ^120^ ^]^ macroporous structure can be created and tailored by advanced fabrication techniques within bulk hydrogel scaffolds. Techniques such as solvent casting, freeze‐drying, phase separation, and gas foaming are frequently utilized to generate macroporosity.^[^ ^121^ ^]^ The advent of 3D bioprinting technology has further simplified the process of introducing macropores into scaffold structures. Besides basic functions in regulating nutrient accessibility, gaseous exchange, and waste elimination in cell‐seeded matrix, these macropores offer several benefits, including ample space for cell growth and the circumvention of transport limitations associated with oxygen, nutrients, and growth factors. Additionally, through regulating cell morphology and assembly, they supply biophysical factors that modulate cellular behavior, and facilitates precise spatial control of pore distribution and interconnectivity, which is crucial for mimicking the native ECM and maintaining cell activities.^[^ ^122^ ^]^ This level of control enables researchers to fine‐tune the hydrogel scaffolds to better replicate the physiological environment, thus leading to more successful in vitro/vivo experiments.

#### 3.2.3 Topography

Native tissue microenvironments, such as the bone, blood vessel, cartilage, and nerve, have highly distinct surface or spatial topography patterns that serve as contact guidance to direct cell responses. According to their scale, topography structures can be classified as micro‐ or nanotopography (Figure 3B). Microtopography, in general, refers to topography features larger than 10 µm in size that influences the entire cell morphology.^[^ ^123^ ^]^ In this respect, the actin cytoskeleton of a cell is responsible for sensing its surrounding structure microtopographical cues, to which it constantly adapts and remodels.^[^ ^124^ ^]^ On the other hand, nanotopography is measured on a nanometer scale and commonly induces changes in cells via subcellular sensory components like integrin and its associated proteins and signaling pathways.^[^ ^123^ ^]^ Many studies have shown that topographical features of engineered hydrogels such as sizes, thickness, patterns, and geometry, play important roles in regulating cell behaviors such as adhesion, migration, and differentiation. Despite the lack of clarity regarding the exact mechanism by which topographical clues are detected, it is believed that a mechanotransduction mechanism similar to normal mechanical stimuli, such as stiffness, plays an indispensable role in this regard. The mechanosensing of topography is unique as the topography is thought to impose a 3D spatial restriction to cell‐hydrogel adhesion sites.^[^ ^125^ ^]^ As a result, the locations, intervals, and sizes of focal adhesion complexes for cell–matrix interaction are fitted to the spatial constraint imposed by the surface architecture. Variations in mechanical signals arising from spatial restriction are transmitted through integrins, and collectively stimulate intracellular signal cascading which leads to phenotypic transformation. To introduce topographical patterns on hydrogel scaffolds, micro‐ and nanopatterning technologies such as photolithography, electron beam lithography, soft lithography, and hot embossing are frequently adopted.^[^ ^126^ ^]^

### 3.3 Dynamic Mechanical Stimulation of Hydrogel Materials

Aside from the hydrogel mechanical properties (i.e., static mechanical stimuli) mentioned above, cells residing in the native ECM experience dynamic mechanical stimulation such as mechanical stretching, compression, torsional force, and flow‐induced shear stress. Of late, it has been established that dynamic mechanical stimulation influences virtually all aspects of cell fates including proliferation, cell morphology, differentiation, and apoptosis, with the resulting effects varying depending on cell type, mechanical forces, and substrate conditions.^[^ ^35^ ^]^ With more evidence supporting the need for appropriate dynamic mechanical stimulation in the study of mechanotransduction, it is critical to provide cells with spatial and temporal mechanical cues similar to those found in their natural environment.^[^ ^31^ ^]^

#### 3.3.1 Mechanical Stretching/Compression (Stress)

Pneumatic actuations, motor‐driven methods, and magnetic actuations are common actuation methods used together with hydrogel systems to deliver local and global mechanical stimuli to cells (Figure 3C).^[^ ^35^ ^]^ Depending on the setup, pneumatic actuation or motor‐driven stretcher/compressor can be used to apply cyclic stretching to adherent cells seeded on/in a stretchable matrix through positive or negative pressure.^[^ ^127^, ^128^ ^]^ This mechanical actuation can also cooperate with the aligned fiber structure, to synergistically regulate cell activities.^[^ ^129^ ^]^ Despite its limitation of inhomogeneous strain distribution, simple configuration of pneumatic and motor‐driven actuation has enabled their widespread use in mechanobiology research, particularly with the commercially available Flexcell system, which yields improved strain profile.^[^ ^130^ ^]^ In recent years, magnetic actuation is one of the most actively researched actuation methods due to its ability to induce well‐controlled mechanical stimulation without physically contacting the culture system.^[^ ^131^, ^132^ ^]^ Magnetic beads or nanoparticles, when combined with a magnetic tweezer, can produce localized mechanical stimuli on the subcellular scale, most notably on the cell plasma membrane.^[^ ^133^ ^]^ Given the noncontact nature of magnetic actuation, this strategy holds promise for in vivo mechanotherapy. Additionally, global deformation of cell‐seeded substrate is attainable by integrating magnetic particles into micropillars, microcantilevers, or continuous soft substrate surfaces at a scale large enough to change whole cell morphology. Finally, magnetoresponsive hydrogel is an emerging strategy for dynamic mechanical stimulation of cells. The encapsulated magnetic beads or rods are attracted and controlled by an external magnetic field which exerts stress on surrounding cells.^[^ ^132^ ^]^ Some of its advantages include high controllability, uniform force distribution reversible/flexible ON/OFF stimulation and noninvasiveness.

#### 3.3.2 Flow‐Induced Shear Stress

Poly(dimethyl siloxane) (PDMS) microfluidic devices coupled with hydrogel‐based matrix are an excellent cell culture platform capable of generating various dynamic mechanical stimulation under well‐controlled conditions. Microfluidic devices, for example, are being used to provide quasi‐circumferential strain,^[^ ^134^ ^]^ hydrostatic pressure,^[^ ^135^ ^]^ and flow‐induced shear stress,^[^ ^136^ ^]^ in order to simulate the physiological conditions of blood vessels and other tissues that are constantly stretched, compressed, or sheared (Figure 3D). Notably, in addition to their versatility and scalability, microfluidic devices can be modified to provide simultaneous biochemical signals or secondary mechanical forces in addition to primary stimulation, allowing for combinatorial effects to be studied. Another crucial aspect is that there is currently a lack of research examining the effects of flow‐induced shear stress on cells encapsulated within hydrogels. Rather, many studies use hydrogels as 2D or 2.5D (2D with a topographical surface which can partially change cell membrane curvature^[^ ^137^ ^]^) substrates, seeding cells onto hydrogel channels or substrates to investigate the impact of flow stress on cell behaviors. Endothelial cells, for example, experience fluid shear stress due to tangential friction between the basal surface of the endothelium and blood or lymphatic flow.

#### 3.3.3 Dynamic Stiffening/Softening

The local properties of ECM are not constant, and their stiffness varies over time, influencing resident cells.^[^ ^138^ ^]^ Over the last few decades, there has been an increase in interest in how ECM stiffness variation influences a plethora of cellular biological behaviors ranging from cell proliferation to metabolism and even pathological activities.^[^ ^114^, ^115^, ^139^, ^140^ ^]^ For example, as the tumor progresses, the ECM and surrounding tissue stiffen gradually due to changes in the structure and composition of the matrix.^[^ ^141^ ^]^ Stiffness variations occur as a result of tissue/organ remodeling, such as break down and reformation of the ovarian matrix during menstruation. To investigate this, new dynamic hydrogel platforms have been developed to modulate the rigidity of the culture environment in a time‐dependent manner by manipulating external stimuli. Leveraging on biochemical factors (e.g., light and enzymatic stimuli for secondary crosslinking) or/and physical stimuli (e.g., pH, temperature, electricity, magnetic field for strain‐stiffening/softening), the matrix stiffness can achieve programmable increase or decrease, or even reservable and flexible stiffness variation for cell mechanotransduction studies.^[^ ^26^ ^]^

#### 3.3.4 Dynamic Viscoelasticity

In addition to stiffness, the ECM exhibits dynamic viscoelastic properties, such as stress relaxation and creep, which result in time‐dependent responses to deformation or mechanical loading. Recent research has increasingly recognized the significance of matrix viscoelasticity in cellular activities and disease progression.^[^ ^142^, ^143^, ^144^, ^145^ ^]^ As a result, hydrogels with adjustable viscoelastic properties show enormous potential for replicating time‐dependent mechanics observed in native ECM, through which they can effectively regulate cellular behavior and direct cell fate. For a more comprehensive understanding of this topic, readers are encouraged to refer to other published review papers.^[^ ^146^ ^]^

## 4 Engineering Hydrogel for Cellular Mechanical Stimulation

Mechanical stimuli, such as hydrogel's inherent matrix properties or externally applied forces play an important role in modulating cell fates and behaviors. Mechanical cues can be used to activate cells and stimulate tissue growth, which is for new therapeutic approaches.^[^ ^147^, ^148^, ^149^ ^]^ Hydrogels are ideal in vitro platforms for cell mechanobiology due to their ECM‐like structure and great biocompatibility.^[^ ^10^, ^150^, ^151^, ^152^ ^]^ Unlike traditional 2D/planar substrates, 3D architecture enables the hydrogel to provide more physiologically relevant conditions and to perform multidimensional mechanical stimulations on cells. More importantly, when used as surface attachments (e.g., wound site) or in vivo implants, these hydrogels can function as programmable cell‐loaded matrix or mechano‐bioreactor through external stimulation.^[^ ^153^ ^]^ Notably, due to cell specificity, a single mechanical stimulus can result in markedly different behaviors in different cell types. This section will focus on hydrogel engineering to mechanically modulate cell types such as mesenchymal stem cells (MSCs), fibroblasts, epithelial cells and immune cells, which have been approved by the U.S. Food and Drug Administration (FDA) for therapeutic use. It also includes endothelial cells as a model cell type for understanding the impacts of shear forces on cells. In each subsection, we first present a stacked bar graph to demonstrate the disparity between static and dynamic mechanical stimuli (based on publication and citation numbers). We aim to draw attention to the paucity of research on the effects of dynamic mechanical stimuli on cells, highlighting the impact it could have on basic mechanobiology studies, cell manufacturing and mechanomedicine, and encouraging more interest in this area.

### 4.1 Mesenchymal Stem Cell

**Figure 4:**
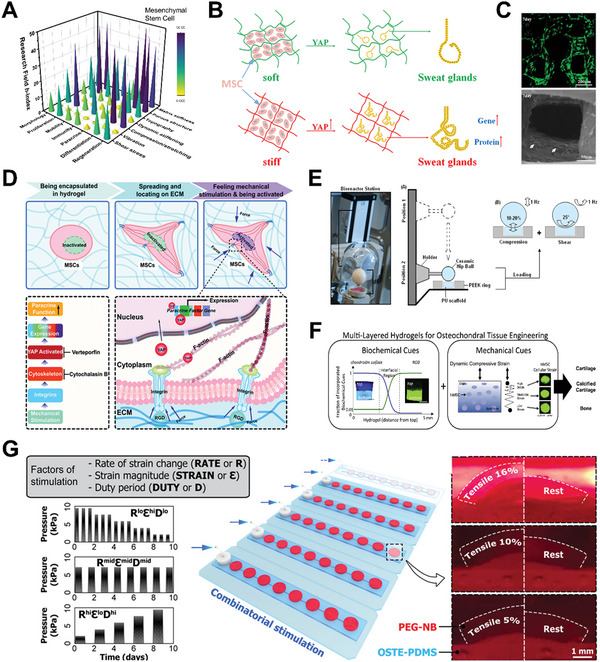
Mechanical stimulation on mesenchymal stem cell (MSC) behaviors by hydrogel‐based platforms. A) 3D bar graph of mechanical stimulation on MSC for various cell activities and biomedical applications. The research conditions are evaluated based on h‐index of each field calculated by paper publication and citation data from *Scopus*. B) Schematic illustration of bioprinted stiffer hydrogels that can upregulate Yes‐associated protein (YAP) level and heighten the expression of sweat gland cell phenotype. Reproduced with permission.^[^ ^178^ ^]^ Copyright 2020, Elsevier. C) Fluorescent and SEM images of MSC (green fluorescence or white arrows) spreading and growing in macroporous silk‐gelatin 3D hydrogel scaffold. Reproduced with permission.^[^ ^122^ ^]^ Copyright 2021, Elsevier. D) Schematic illustration of mechanical stimulation from dynamic stiffening matrix. The stiffness stress is transmitted via cell cytoskeleton and induces YAP nuclear localization and ultimately potentiates paracrine response. Reproduced with permission.^[^ ^82^ ^]^ Copyright 2020, the Royal Society of Chemistry. E) Schematic illustration of bioreactor operation for compression and shear forces to MSC‐seeded hydrogel. Reproduced with permission.^[^ ^195^ ^]^ Copyright 2017, Springer Nature. F) Multilayer hydrogel with specific biochemical cues, matrix stiffness, and dynamic mechanical loading for osteochondral tissue engineering. Reproduced with permission.^[^ ^197^ ^]^ Copyright 2015, Elsevier. G) Illustration and images of combinatorial mechanical stimulation patterns in combination with defined levels of rate of strain change, strain magnitude, and duty period. Reproduced with permission.^[^ ^190^ ^]^ Copyright 2021, American Association for the Advancement of Science.

MSCs are fibroblastoid, multipotent adult stem cells that can be found in a variety of bone and adipose tissues. MSCs possess high cell proliferation, self‐renewal ability, and are sensitive to the surrounding environments.^[^ ^82^ ^]^ Under specific biophysical or/and biochemical stimulation, MSCs can differentiate into adipocytes, osteoblasts, and chondrocytes, or transdifferentiate into non‐mesenchymal cell lineages and mesodermal cell lineages.^[^ ^154^, ^155^, ^156^, ^157^, ^158^ ^]^ Their remarkable self‐renewal capabilities and lineage differentiation have drawn considerable attention in the realm of regenerative medicine. As of December 2022, there are over 1400 registered clinical trials using MSCs to investigate the therapeutic effect, including bone/cartilage repair, immunoengineering, and even the prevention or treatment of COVID‐19, listed on ClinicalTrials.gov.^[^ ^159^ ^]^ Conventionally, culture medium with biochemical factors is used to induce MSC differentiation into targeted cell types. Nonetheless, this approach necessitates the addition of fresh differentiation culture medium on a regulate basis to avoid premature cell aging as the number of passages increases, and it is not suitable for in vivo differentiation for clinical purposes.^[^ ^160^, ^161^ ^]^ Recent studies have highlighted the potential of harnessing mechanoactivation to trigger MSCs proliferation, differentiation, and paracrine activities (e.g., growth factor and exosome) for clinical application including inducing MSCs‐derived bone tissue regeneration.^[^ ^162^, ^163^, ^164^, ^165^ ^]^ By programming physical properties of hydrogel scaffold (e.g., matrix stiffness and internal topography) and applying dynamic forces, researchers have discovered the widespread implications of mechanical stimuli on cell biofunctions (**Figure** **4**A).

#### 4.1.1 Internal Topography

Engineering geometric architectures has proven to be a successful method for regulating MSC fate. The shape and size of MSCs can be easily controlled by tuning matrix internal or surface microstructures.^[^ ^166^ ^]^ Generally, a small and roundish matrix structure limits MSC flattening and hence promotes differentiation toward adipocytes lineage, whereas a larger matrix structure promotes cell spreading and thereby favors osteoblastic differentiation.^[^ ^167^, ^168^ ^]^ By capitalizing on electrospinning and other advanced hydrogel fabrication methods, various fibrous architectures can be fabricated in 3D scaffolds to mimic MSCs–matrix interaction. Several MSC studies using fibrous hydrogel have illustrated the effects of scaffold architecture on MSC activities, particularly cell proliferation, spreading,^[^ ^169^ ^]^ and chondrogenic differentiation.^[^ ^170^ ^]^ Furthermore, porous structure is another important geometric feature that influences MSC behaviors. Hydrogel macroporosity has been found to be directly implicated MSC growth under 3D conditions. In vitro, human MSCs seeded in porous hydrogel showed improved oxygenation and osteogenic lineage commitment, as well as early expression of alkaline phosphate and collagen type I.^[^ ^171^ ^]^ Immunostaining revealed that the expression of HIF‐*α* in solid hydrogels was significantly higher than in porous hydrogels, indicating a greater degree of oxygen deprivation. When the macroporous hydrogel construct was transplanted into mice, it induced vascularization and promoted oxygenation of the embedded cells.

#### 4.1.2 Stiffness

Matrix stiffness has a strong influence on stem cell differentiation. There is abundant evidence demonstrating that matrix stiffness regulates MSC differentiation toward bone, muscle or neuronal lineages when they are seeded on hydrogel matrices that are similar to the native stiffness of their respective tissues.^[^ ^172^, ^173^ ^]^ MSCs cultured on soft matrix preferentially undergo adipogenesis and chondrogenesis, whereas those cultured on stiffer matrix preferentially undergo osteogenesis.^[^ ^143^, ^174^ ^]^ Evidently, stiff substrate induces higher expression of smooth muscle cell markers, while soft substrate promotes the expression of chondrogenic markers but results in less cell spreading, fewer stress fibers, and lower proliferation rate.^[^ ^175^ ^]^ The tension and its resulting cytoskeleton contractability, as well as the adhesion site transduction are considered the main factors contributing to the stiffness‐dependent differentiation (Figure 2).^[^ ^176^ ^]^

Similar to bulk hydrogels, the stiffness of 3D‐bioprinted matrix can affect MSCs differentiation as well. In recent years, tunable hydrogel‐based bioink and bioprinted matrices have been widely used in MSCs‐related bone engineering. For example, by leveraging on 3D‐printed hydrogel, Liu et al. observed a higher expression of osteogenic markers in stiffer hydrogels, indicating a stronger response of MSCs differentiation toward stiffer matrix.^[^ ^177^ ^]^ Following that, they discovered that the stiffness of hydrogel‐based bioink can also regulate MSC differentiation toward sweat gland cells, potentially by upregulating YAP localization in the nuclei (Figure 4B).^[^ ^178^ ^]^ 3D bioprinting can thus be utilized to configure microenvironments (i.e., stiffness‐gradient constructs) and direct desired MSC phenotypes.^[^ ^179^ ^]^ Additionally, 3D bioprinting can introduce macropores within scaffold structure, which can provide adequate space for cell growth and overcome the transport restriction for oxygen, nutrient, and growth factors (Figure 4C).^[^ ^122^ ^]^

Matrix stiffness is also a potential modulator of redox metabolism in MSCs. Tay and co‐workers observed increased expression of intracellular ROS expression on softer substrates through the mechanotransduction pathway.^[^ ^180^ ^]^ Consequently, the production of proangiogenic transcriptors was upregulated, as evidenced by higher expressions of vascular endothelial growth factor (VEGF) and basic fibroblast growth factor on softer matrix. This finding emphasizes the possibility of reprogramming MSCs redox metabolism by modifying material mechanical features for regenerative medicine using MSC‐derived secretome. In addition to MSCs, the proliferation and differentiation of some other stem cells such as adipose‐derived stem cells are reportedly affected by hydrogel matrix stiffness as well.^[^ ^181^ ^]^

#### 4.1.3 Dynamic Stiffening

Aside from local static matrix stiffness, stiffness variation/gradient is another important modulator of MSC behaviors. MSC migration and differentiation can be directed by stiffness gradients within ECM or tissues caused by normal tissue variation (e.g., myocardium) or pathological conditions (e.g., myocardial infarction).^[^ ^182^ ^]^ MSC cellular adhesion and spreading increased in a stiffness gradient gel via F‐actin assembly and vinculin recruitment.^[^ ^183^ ^]^ Even with a shallow durotactic gradient, MSCs eventually migrate to the stiffer region and differentiate into a more contractile and myogenic phenotype, demonstrating the importance of mechanical tension in MSC growth and differentiation.^[^ ^117^ ^]^ Changes in ECM stiffness also affect the paracrine function of MSCs, including VEGF, SDF‐1, and HGF. Compared to static stiffness, dynamic stiffening of the matrix is a more effective mechanical stimulation to activate encapsulated MSCs. By leveraging on the release of biomolecules, Lin et al. discovered that by using calcium ions release to stiffen alginate‐RGD hydrogel, MSCs displayed stronger YAP activation and paracrine function than those in hydrogel without dynamic stiffening.^[^ ^82^ ^]^ This is because the soft matrix allows for early cell spreading, resulting in a stable cytoskeleton that helps MSCs to collect more mechanical signals when the matrix stiffens (Figure 4D).

It is noteworthy that static mechanical stimuli promote certain cell behaviors while limiting some others,^[^ ^184^ ^]^ whereas dynamic stiffening can effectively regulate multiple cell activities across time through programmable material. This feature is beneficial for improving therapeutic outcomes in clinical applications as well as increasing biomolecule production. For example, it was discovered that MSCs cultured on soft gel substrate significantly improved the secretion of immunomodulatory factors but halted MSC proliferation, while stiff substrate resulted in enhanced proliferation and reduced replicative senescence without any secretome amplification. Hence, a novel approach would be to first introduce stiff substrate to promote MSC proliferation and follow it up with gel softening to improve secretory activities to produce a greater quantity of therapeutic secreted factors (e.g., VEGF). In addition to mechanical cues, dynamic stimulation can be relevant also to biochemical cues, such as changing ligand presentation on demand. Bian's group developed a dynamic hydrogel platform that provides magnetic tuning of RGD tether mobility within the hydrogel network, allowing stem cell behaviors to be regulated.^[^ ^185^ ^]^ Furthermore, they also discovered that cancer cells with increased stemness and tumorigenicity exhibit dynamic controllable presentation of integrin ligands. This discovery emphasizes the potential impact of modulating ligand presentation on cell behavior and its implications for cancer research.^[^ ^186^ ^]^ This demonstrates the importance of modulating the biochemical presentation of cell ligands on hydrogel/matrix for dynamic control of cell–matrix interactions, which has significant value for both fundamental research and practical applications.^[^ ^3^ ^]^ Readers are encouraged to refer to published reviews elsewhere.^[^ ^187^ ^]^

#### 4.1.4 Dynamic Loading

Applying external stress is another way of manipulating the dynamic mechanical features of MSC’s surrounding microenvironment. It was previously found that the effect of static matrix stiffness or constant mechanical stimulation gradually faded due to cell adaptation.^[^ ^188^, ^189^ ^]^ The application of dynamic loading is believed to alleviate the effects of cell adaptation by resetting cell mechanosensitivity and enhancing cellular mechanoresponses.^[^ ^190^, ^191^, ^192^ ^]^ Through the use of an external bioreactor, incremental or cyclic strain is commonly applied to cell‐laden matrix as dynamic mechanical stimuli to regulate MSC behaviors. In particular, compression loading has been shown to enhance MSC chondrogenic differentiation, which is required for neocartilage formation.

Compressing MSC‐seeded hydrogel with TGF‐1 increased chondrogenic gene expression (i.e., Sox‐9, aggrecan, and collagen type II) and ECM production.^[^ ^193^ ^]^ Specifically, a duration of 2.0–2.5 h appeared to be optimal for chondrogenic differentiation. A shorter duration is inadequate to stimulate the encapsulated cells whereas a longer duration causes cell–cell signaling to level off, limiting the stimulation effect. In addition, this study also highlighted the significance of TGF‐*β* to MSC chondrogenesis, and that dynamic compression may have enhanced the transport of TGF‐*β* into the hydrogel.^[^ ^194^ ^]^ Nevertheless, some other studies have demonstrated that external force alone, without exogenous biochemical factors, can significantly induce and maintain MSC chondrogenesis. For example, a combination of compression and shear forces for 21 days in a hydrogel bioreactor (Figure 4E) without exogeneous factors can facilitate chondrogenic differentiation with an increased chondrogenic gene and protein expression (e.g., sulphated glycosaminoglycans and collagen II).^[^ ^195^ ^]^

The benefits of dynamic mechanical stimulation shed light on the field of MSCs‐based tissue engineering including cartilage regeneration or repair. As such, many researchers have explored techniques like in vitro biochemical/biomechanical chondrogenic preconditioning and in situ MSCs‐laden scaffold implants. For example, Lin et al. implanted MSCs‐seeded hydrogel treated with chondrogenic induction medium and 14 days of dynamic compressive loading into osteochondral‐defected animal models and discovered that the mechanical condition promoted neocartilage formation.^[^ ^196^ ^]^ Steinmetz et al. used a sequential photopolymerization method to develop a multilayer hydrogel that comprises a soft, cartilage‐like layer of chondroitin sulfate with low RGD concentrations, a stiff bone‐like layer with high RGD concentrations, as well as an intermediate interfacial layer for cartilage and bone compound tissue regeneration.^[^ ^197^ ^]^ Compared to MSCs cultured statically in differentiation media, dynamic mechanical stimulation increased the expression of collagen type II in the cartilage‐like layer, collagen type X in the interfacial layer, as well as collagen type I in the bone‐like layer and mineral deposits localized to the bone layer (Figure 4F). Aside from compression, interstitial flow is another potential factor triggering osteogenesis in stiff bone‐like layer, as previous studies has shown that flow‐induced shear stress may potentiate the osteogenic differentiation of MSCs via TAZ activation.^[^ ^198^ ^]^ In these works, the static preculture (commonly culture several days without mechanical stimulation) is required before applying dynamic loading. In the absence of preculturing, compression‐induced MSC chondrogenic differentiation was reduced due to the poor adaptation to the new matrix environment and insufficient acquisition of nutrient or biochemical signals.^[^ ^199^, ^200^ ^]^ Similar to compression, stretching or tensile stress can influence MSC fate. Liu et al. discovered that increasing the duty period and strain magnitude synchronously can most effectively improve MSC matrix production (Figure 4G).^[^ ^190^ ^]^

#### 4.1.5 Vibration

Vibration has been reported to promote ontogenetic differentiation of MSC via mechanotransduction pathways. Vibration is exerted on muscles and bones during daily physical activities such as walking and running. This phenomenon has been shown to affect bone mass and skeleton strength.^[^ ^161^, ^201^, ^202^ ^]^ As a result, researchers begin to take advantage of the unique property of vibration to promote MSC activities, particularly osteogenesis. For example. Mehta et al. encapsulated hMSCs in PEGDA gel microspheres and stimulated them with low‐magnitude, high‐frequency vibration. They discovered that low (0.3 g) and medium (3.0 g) accelerations enhanced osteogenesis in MSCs while high accelerations (6.0 g) inhibited osteogenesis due to cell apoptosis and reduced bone resorption.^[^ ^173^ ^]^

The native mechanical microenvironment of MSCs is highly intricate and contains multiple mechanical cues that have to be coordinated for delicate control of MSC activities and functions.^[^ ^203^, ^204^ ^]^ Systematic and combinatorial approaches, which included multiple static or dynamic mechanical stimulation (i.e., mechanical cocktail), provide more predictive regulation of MSC mechanoresponses. For example, Grolman et al. demonstrated that ECM plasticity and dynamic force loading can affect MSC phenotypes and spreading through a biphasic relationship dependent on cell intrinsic forces.^[^ ^205^ ^]^ Despite the fact that a large number of external devices for force‐loading purposes have been developed, translating them into clinical applications involving osteo‐chondrogenesis remains difficult. In this regard, magnetic and ultrasonic mechanical stimulation are attractive strategies due to their remote actuation and noninvasive nature.^[^ ^35^ ^]^ Another major obstacle to current MSC‐based research is its inconsistent experimental or clinical results. Currently, several studies have illustrated the effects of mechanical stimulation, but these results often appeared to be inconsistent, probably caused by differences in cell source species (i.e., mice and human), cell types/subpopulation (MSC is a highly heterogeneous cell population with multiple subsets), and culture conditions.^[^ ^166^ ^]^ It is therefore important to optimize and standardize MSC source and culture conditions to improve the reproducibility of experiments. This is also meaningful for future MSC autologous cell extraction and therapy in the clinic. Some other features including seeding cell density,^[^ ^206^ ^]^ cell–matrix interaction (e.g., ECM reconstruction),^[^ ^36^, ^82^ ^]^ synergistic promotion with biochemical factors (e.g., TGF‐*β*),^[^ ^175^ ^]^ and hydrogel crosslinking rate,^[^ ^207^ ^]^ are associated with the effect of MSC mechanobiological‐based control.

### 4.2 Fibroblast

**Figure 5:**
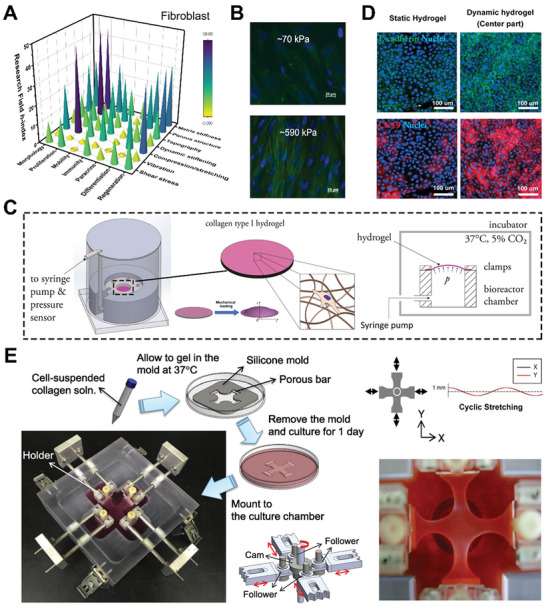
Mechanical stimulation on fibroblast cell behaviors by hydrogel‐based platforms. A) 3D bar graph of mechanical stimulation on fibroblast for various cell activities and biomedical applications. The research conditions are evaluated based on h‐index of each field were calculated by paper publication and citation data from *Scopus*. B) Fluorescent staining indicates that fibroblasts, which cultured in matrix with higher stiffness, express more *α*‐smooth muscle actin (*α*‐SMA). The cells are detected by staining the cell nucleus (blue DAPI) and *α*‐SMA (fluorescent green). Reproduced with permission.^[^ ^213^ ^]^ Copyright 2012, Elsevier. C) Schematic illustration of dynamic pump‐based bioreactor that applies tension to deform installed hydrogels. Reproduced with permission.^[^ ^227^ ^]^ Copyright 2021, Elsevier. D) Whole‐mount staining for E‐cadherin and CK19 expression suggests that dynamic forces can promote the early maturation of the dermo–epidermal skin substitute. Adapted with permission.^[^ ^227^ ^]^ Copyright 2021, Elsevier. E) Schematic and working procedure of the cyclic four‐arm stretcher. The fibroblast‐seeded hydrogel is installed in the chamber and stretched by dynamic actuator system. Adapted with permission.^[^ ^231^ ^]^ Copyright 2018, Elsevier.

Fibroblast is a type of mesenchymal cell that constitutes a majority of the stroma, especially in connective tissues. They are typically spindle‐shaped, elongated morphologies with a flat oval nucleus. As of December 2022, there were over 1100 registered clinical trials using fibroblasts to investigate the therapeutic effect, the majority of which most were for skin regeneration or transplantation for a variety of disease/indications such as wrinkles correction (LAVIV, Rosmir), diabetic foot ulcer (DERMAGRAFT), mucogingival condition (GINTUIT), and deep partial‐thickness burns (STRATAGRAFT). For example, DERMAGRAFT, a fibroblast‐derived dermal substitute which is composed of fibroblasts, ECM, and a bioabsorbable scaffold, is approved for the treatment of full‐thickness diabetic foot ulcers. Inactivated fibroblasts exist in a quiescent state with low secretion and contractile activity. When activated fibroblasts (e.g., proto‐myofibroblast and myofibroblast) are stimulated by extracellular biochemical and mechanical cues, they secrete ECM macromolecules (e.g., collagen, glycosaminoglycan, proteoglycan), growth factors, cytokines, and chemokines.^[^ ^208^, ^209^ ^]^ Fibroblasts can also remodel and compact their surrounding matrix, particularly the collagen construct, via pseudopodia extensions (usually several tens of µm within a matter of hours).^[^ ^210^ ^]^ Material mechanical properties and external dynamic stress have been shown to have a significant impact on fibroblast behavior (**Figure** **5**A).

#### 4.2.1 Stiffness

Many recent studies using hydrogel models have demonstrated that matrix stiffness could regulate fibroblastic cell behaviors and phenotypes. Jiang et al. designed a stiffness‐controllable hydrogel by modifying DNA crosslinking, and they demonstrated that fibroblasts seeded in the material could change morphologies in response to different rigidity.^[^ ^211^ ^]^ Similar results were observed in Yeung et al. where fibroblasts cultured on soft gel were round in shape while those cultured on stiffer gel were of elongated morphologies with articulated stress fibers.^[^ ^212^ ^]^ Chia et al. revealed that upon exposure to TGF‐*β*, fibroblasts cultured on rigid polyethylene glycol (PEG) hydrogels exhibited a higher expression of *α*‐smooth muscle actin (*α*‐SMA) fibers compared to those on soft PEG hydrogels (Figure 5B).^[^ ^213^ ^]^ Interestingly, stiff matrix promoted fibroblast proliferation^[^ ^214^ ^]^ and transdifferentiation into myofibroblast,^[^ ^215^ ^]^ a type of activated phenotype that provides contractile forces during wound closure.^[^ ^216^ ^]^ As a result of this discovery, novel wound healing strategies have been developed and tested. For example, high stiffness hydrogel‐based wound dressing was found to induce fibroblast proliferation, enhance stress fiber formation as well as upregulate key mediators of wound inflammation (e.g., interleukin‐10).^[^ ^217^, ^218^ ^]^ However, stiff matrix‐induced fibroblast/myofibroblast transition may cause fibrotic pathologies including valvular stenosis.^[^ ^219^, ^220^ ^]^ In high‐stiffness matrix (i.e., pathological conditions), fibroblasts can also differentiate into carcinoma‐associated fibroblasts, which is a key component of tumor ECM that contributes to tumorigenesis.^[^ ^221^, ^222^ ^]^

#### 4.2.2 Macroporosity of Hydrogel Network and Scaffold

Macroporous structure of a hydrogel matrix is another important mechanical property that influences fibroblast behavior. In this context, Choi et al. fabricated gelatin hydrogel scaffolds with different macroporous structures through 3D printing technique. Evidently, cells cultured in the larger pores proliferated faster than those in smaller ones.^[^ ^223^ ^]^ This finding suggested that a hydrogel scaffold with the appropriate porous structure could support fibroblast proliferation and migration. Given the close relationship between stiffness and mesh size, it is hypothesized that both properties have synergistic effects on fibroblast cellular behaviors.^[^ ^224^ ^]^ While the pore architecture of hydrogel may have a significant impact on cellular processes, fibroblasts residing within the construct could in turn manipulate the mechanical properties,^[^ ^210^, ^225^ ^]^ particularly the stiffness of hydrogel. Based on Ahearne et al., fibroblasts altered the elastic modulus of the hydrogel by applying contractile forces, resulting in matrix contraction or compaction (i.e., modulus increase) while releasing MMP to reduce gel mechanical strength.^[^ ^226^ ^]^ Following that, changes in the surrounding matrix would have a negative impact on cell movement, viability, and actin expression. A mechano‐feedback loop exists between the fibroblast and the surrounding matrix, in which the mechanotransduction channels and cytoskeleton of fibroblast are strongly affected by the surrounding, while the fibroblasts release biomolecules (e.g., cytokines, chemokines, ECM macromolecules) and exert forces on the matrix.

#### 4.2.3 Dynamic Compression/Stretching

As the key components of connective tissues and organs (e.g., skin), fibroblasts are subjected to a variety of dynamic forces, such as tension from neighboring cells/surrounding matrix and stress from physical exercises. These applied forces play critical roles in regulating the cell behavior of fibroblasts. Researchers have developed a number of devices/systems for applying external forces to hydrogels in order to observe how the applied forces affect fibroblast cell behaviors. For example, Wahlsten et al. created a dynamic bioreactor to introduce cyclic deformation to fibroblast‐seeded collagen hydrogel (Figure 5C).^[^ ^227^ ^]^ The results showed that when fibroblasts were cultured under dynamic mechanical stress, the number of fibroblasts increased by 75%, and the direction of hydrogel deformation influenced fibroblast orientation. Similar results were found in the work of Balestrini and Billiar, which discovered that intermittent stretching (i.e., dynamic mechanical force) applied on fibroblast‐populated fibrin gel could increase cell number due to increased mechanotransduction pathway and collagen secretion under hydrogel compaction, while continuous force (i.e., static mechanical force) had no significant impact.^[^ ^228^ ^]^ More importantly, they demonstrated that dynamic mechanical loading on fibroblast‐seeded matrix could promote the production of human dermo–epidermal skin substitutes (Figure 5D), which could be used in skin graft and reconstructive surgery. Hu's group developed a four‐arm hydrogel stretcher that uses biaxial/uniaxial mechanical constraints to stimulate fibroblast‐seeded construct (Figure 5E).^[^ ^229^, ^230^, ^231^ ^]^ In the presence of cyclic biaxial stretching, fibroblasts had an elongated, spindle‐shaped morphology with increased *α*‐SMA expression, whereas cells seeded in the unstretched gels had a spherical morphology with low *α*‐SMA expression. This mechanoactivation also has an important contribution in ECM remodeling and tissue development. Lee et al. demonstrated that incrementally increasing stretching significantly increased the mRNA expression of collagen type I.^[^ ^231^ ^]^ Nevertheless, because a force threshold affects fibroblast collagen production, the applied force should be within the appropriate range.^[^ ^232^ ^]^

The aforementioned studies provide new insight into the behaviors of mechano‐stimulated fibroblasts. However, some limitations must be addressed in order to promote the clinical translation of fibroblastic mechanotherapy. However, external mechanical stimulation has both positive and negative effects on cellular behaviors, which is a significant limitation. A recent study revealed an inverse relationship between fibroblast proliferation and collagen synthesis, implying that cyclic force enhanced collagen production but could possibly hinder cell proliferation simultaneously.^[^ ^233^ ^]^ In addition, inappropriate mechanical forces may jeopardize the mechanical landscape of ECM by increasing tissue stiffness, resulting in abnormal cellular behaviors. Hence, to achieve the desired mechanostimulation with minimal side effects, the hydrogel mechanical characteristics and magnitude/range of mechanical forces applied on targeted fibroblasts must be quantified precisely. Although some crucial parameters such as stretch parameters^[^ ^228^ ^]^ and matrix (i.e., hydrogel) remodeling metrics^[^ ^234^ ^]^ have been investigated thoroughly, more holistic and comprehensive research is required for further clinical validation. Furthermore, it is critical to understand the complex relationship between various mechanical stimuli, including the synergistic effects and dominant factors that determine cellular behaviors. As shown in Figure 5A, current mechanobiology research on fibroblasts focuses a lot on material static mechanical features, especially on stiffness. However, Wahlsten et al. discovered that cyclic stress on hydrogel is the dominant mechanical factor promoting fibroblast proliferation, whereas hydrogel stiffness only plays a relatively minor role.^[^ ^227^ ^]^ Similarly, Petersen et al. discovered that gel scaffold stiffness has only a transient impact on fibroblast cell behaviors, whereas the effect of mechanical stimulation was preserved over time.^[^ ^235^ ^]^ Henceforth, more emphasis should arguably be placed on externally applied forces for the application of fibroblast in skin tissue engineering. Interestingly, many mechano‐induced cell behaviors and mechanisms remain unknown. As an example, it is still unclear how different mechanical stimuli trigger fibroblasts to differentiate into myofibroblasts and more research is needed to elucidate the specific mechanisms.

### 4.3 Epithelial Cell

**Figure 6:**
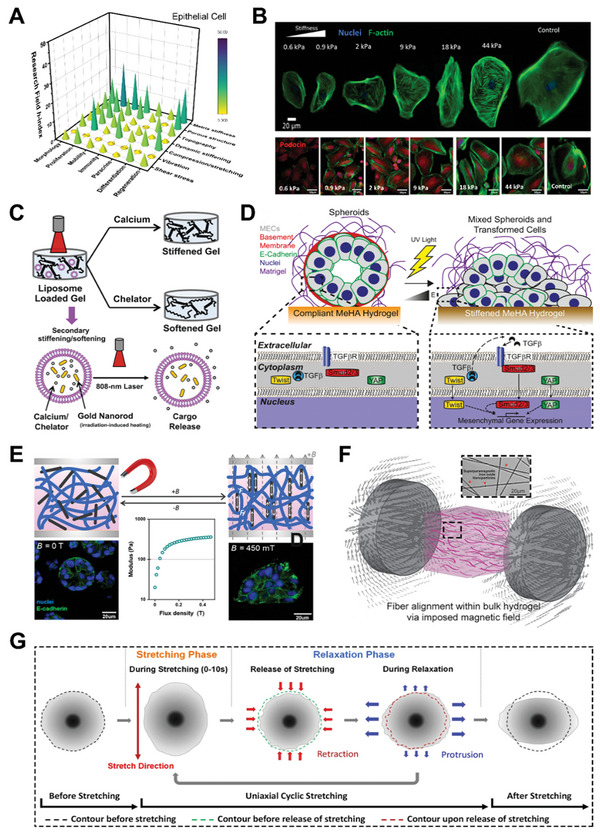
Mechanical stimulation on epithelial cell behaviors by hydrogel‐based platforms. A) 3D bar graph of mechanical stimulation on epithelial cells for various cell activities and biomedical applications. The research conditions are evaluated based on h‐index of each field were calculated by paper publication and citation data from *Scopus*. B) Immunofluorescence images of podocytes cultured on polyacrylamide hydrogels which elasticity ranging between 0.6 and 44 kPa and have different actin cytoskeleton architecture and podocin levels. Cell nucleus, actin cytoskeleton, and podocin protein are stained by blue DAPI, green phalloidin, and red antipodocin dye, respectively. Reproduced with permission.^[^ ^243^ ^]^ Copyright 2019, American Chemical Society. C) Schematic illustration of dynamic irradiation stiffening/softening hydrogel. The nanorods generate heat under irradiation and result in calcium/chelator releasing from liposome. Adapted with permission.^[^ ^248^ ^]^ Copyright 2015, National Academy of Sciences. D) Mechanism illustration of matrix (gel) stiffness‐modulated mammary epithelial cell spreading through mechanotransduction (TGF‐*β* and YAP). Adapted with permission.^[^ ^251^ ^]^ Copyright 2019, National Academy of Sciences. E) Schematic illustration of hydrogel magnetic stiffening and representative immunofluorescence images of induced epithelial cell morphology switch. Reproduced with permission.^[^ ^253^ ^]^ Copyright 2021, American Chemical Society. F) Schematic illustration of internal fiber alignment modulation by external magnetic field, and transmitted light image of superparamagnetic iron oxide nanoparticles (red arrows) within electrospun dextran vinyl sulfone fibers. Reproduced with permission.^[^ ^254^ ^]^ Copyright 2021, Frontiers Media S.A. G) Schematic illustration of the protrusion/retraction activities during relaxation phase which cause cell reorientation in response to uniaxial cyclic stretching. Reproduced with permission.^[^ ^258^ ^]^ Copyright 2021, Springer Nature.

Most body/organ surfaces are covered by epithelial cells, which form physical tissue barriers known as the epithelium. There are three main types of epithelial cells: squamous (flat), cuboidal (cube‐shaped), and columnar (column‐shaped). Different types of epithelial cells could be organized into simple (one‐layer), stratified (multilayer), pseudostratified (one‐layer with different cell sizes), and transitional layers.^[^ ^236^ ^]^ They are the key sentinel of the body's immune system. Moreover, epithelial cells located at different body parts may have distinctive tissue‐specific functions. For instance, epithelial cells are involved in the tissue repair and remodeling process of the airways in collaboration with fibroblasts.^[^ ^237^ ^]^ Keratinocytes are the most common type of epithelial cell (≈95%), and make up the skin's outermost layer (usually referred to as epithelial skin cells).^[^ ^238^, ^239^ ^]^ They are widely utilized in regenerative medicine including skin/scalp epidermis repair, skin substitutes, and wound healing.^[^ ^240^, ^241^ ^]^ As of December 2022, there are over 200 registered clinical trials for keratinocytes worldwide listed on ClinicalTrials.gov, and some of them have already been approved by FDA (e.g., STRATAGRAFT, an allogeneic cellular product with keratinocytes and dermal fibroblasts in collagen scaffold indicated for deep partial‐thickness burns). It has been shown that epithelial cells are influenced by various mechanical stimuli derived from both matrix physical features (i.e., the surrounding environment) and external dynamic forces. For example, researchers discovered that intercellular or external mechanical cues via RhoA activity drive the development of leading cells (i.e., epithelial cells at the edge of migration fingers) during epithelium collective migration.^[^ ^242^ ^]^ To investigate epithelial mechanoresponsive behaviors further, a series of hydrogel scaffolds are utilized for ECM emulation (**Figure** **6**A).

#### 4.3.1 Stiffness

Several studies have found that the activation of epithelial cells is closely related to their surrounding matrix stiffness, and that different cell types respond distinctively to ECM stiffness. For instance, podocytes, a type of highly specialized glomerular epithelial cell, can detect and respond to changes in substrate stiffness accordingly. Abdallah et al. utilized hydrolyzed PAA hydrogels with elastic modulus ranging from 0.6 to 44 kPa to investigate the rigidity‐dependent behaviors of podocytes.^[^ ^243^ ^]^ They discovered that a stiffer substrate (44 kPa) induced a spreading phenotype with a dense actin cytoskeleton, while podocin was expressed at a higher level at 0.9–9 kPa (Figure 6B), which is consistent with the stiffness of the glomerular basement membrane. This observation could be associated to the activity of adhesion molecules such as integrin.^[^ ^244^ ^]^ Furthermore, matrix stiffness also has a dominating effect on retinal pigment epithelium cells. When relevant exogenous factors such as Activin A were present, White et al. found that matrices with appropriate stiffness (i.e., hydrogel scaffolds with high stiffness) improved cell survival, activities, and functions.^[^ ^245^ ^]^ It was discovered in the same study that stiffening of matrix is strongly correlated to epithelial disease progression by providing a malignant environment. Aside from that, Eisenberg et al. demonstrated that stiffer PAA gel induced phenotypic changes in lung alveolar epithelial cells, as well as increased ECM protein deposition and organization, such as laminin and fibronectin, leading to the progression of fibrotic lung diseases.^[^ ^246^ ^]^ Furthermore, Gill et al. utilized a tunable, synthetic, RGD‐modified PEG hydrogel to investigate epithelial morphogenesis, and it was found that different degrees of matrix stiffening could enhance the epithelial to mesenchymal transition (EMT), lung adenocarcinoma, and tumor metastasis.^[^ ^247^ ^]^

#### 4.3.2 Dynamic Stiffening

However, as matrix stiffening (e.g., normal to malignant stiffness) is an intricate, gradual, and dynamic process, it is impractical to achieve such an event with conventional static hydrogels. In this regard, gradually or temporally stiffening hydrogel materials have been designed. Suggs's group engineered dynamic stiffening/softening alginate/Matrigel composite hydrogel using secondary crosslinking/degradation via light‐triggered release of calcium or calcium chelators (e.g., diethylenetriamine‐penta‐acetic acid) from liposomes (Figure 6C) to mimic the native dynamic process.^[^ ^248^ ^]^ Based on the results, they discovered that during matrix stiffening, nonmalignant mammary epithelial cells lost their epithelial features and transitioned into a mesenchymal invasive phenotype, which is associated with tumor progression and metastasis.^[^ ^249^ ^]^ The EMT‐related changes were revealed to be dependent on several mechanical signaling pathways (e.g., FAK, PI3K) in their subsequent work.^[^ ^250^ ^]^ Ondeck et al. reported similar EMT findings involving more mechanotransduction signaling pathways (TGF and YAP), which used a two‐stage photopolymerization hydrogel to produce dynamic substrate stiffness (Figure 6D).^[^ ^251^ ^]^

Magneticresponsive hydrogel, which has the advantages of easy manipulation, remote control, modulable strength, and deep penetration, is another strategy for achieving dynamic substrate stiffness.^[^ ^252^ ^]^ The gels can respond to the applied external magnetic field by adding magnetic components such as iron particles or magnetite nanorods, allowing the mechanical stiffness to be easily and reversibly tailored. For example, Chen et al. developed a series of ferrogels (polyisocyanide‐based hydrogels with magneticsensitive nanorods) that can be dynamically stiffened and induced internal strain when exposed to an electromagnet.^[^ ^253^ ^]^ During magnetic stiffening, epithelial cell clusters cultured in the ferrogel transformed into invasive morphologies (Figure 6E). Nonetheless, a relatively large amount of magnetic nanorods must be incorporated into the gel to exert physiologically comparable stiffening and adequate stress to cells, which may result in gel microstructural disruption and cytotoxicity. To ensure uniform force distribution, it is also critical to avoid particle aggregation by employing strategies such as mixing and sonication.

#### 4.3.3 Dynamic Fibrous Structure

The internal hydrogel microstructure can also be modulated by magnetic field. Hiraki et al. integrated superparamagnetic iron oxide nanoparticles into dextran‐based fibers during electrospinning to fabricate 3D hydrogel composites that can generate specific fiber alignments by applying an external magnetic field (Figure 6F).^[^ ^254^ ^]^ Interestingly, they noticed that fiber alignment not only affected the direction of epithelial cell migration but also induced cell–cell breakage events, resulting in a spreading phenotype that is beneficial for tendon repair therapy. Stretchable bioreactor platforms can also be used to achieve hydrogel gradual stiffening.^[^ ^255^ ^]^

#### 4.3.4 Dynamic Compression/Stretching

Epithelial cells are constantly exposed to a dynamic mechanical environment in vivo, which influences both physiological and pathological events. The epithelial layer of organs is always subject to some degree of stretching such as that caused by breathing movements, cardiac pulses, as well as skin and muscle contractions.^[^ ^256^ ^]^ These mechanical stretching typically direct epithelial cell behavior through mechanical signaling pathways (e.g., Piezo1).^[^ ^257^ ^]^ In recent years, many new insights have been generated by the use of various hydrogel systems. Lien and Wang observed cyclic stretching‐induced epithelial cell reorientation by using an on‐stage cyclic stretching gel device.^[^ ^258^ ^]^ They outlined the roles of mechanical stretching and relaxation in cell reorientation, highlighting the significance of the relaxation phase in epithelium transverse extension (Figure 6G). Using soft polyacrylamide gel attached to a stretchable PDMS membrane, Casares et al. demonstrated that the tissue stretching‐induced epithelial fracture was largely caused by hydraulic pressure and could be easily healed via actomyosin‐dependent mechanisms.^[^ ^259^ ^]^ Using hydrogel models, epithelial behaviors and interaction/cooperation with other cells such as fibroblast and endothelial cells can be studied in the presence of mechanical stretching.^[^ ^227^, ^260^ ^]^ Notably, samples subjected to mechanical stretching yield significantly higher EMT effects than statically samples. These results imply that dynamic mechanical stress has a greater stimulation effect than static mechanical stress. Mechanical stretching aside, more research is required to determine how dynamic mechanical stimulation can supplement the next generation of epithelial regenerative medicine.

### 4.4 Immune Cell

**Figure 7:**
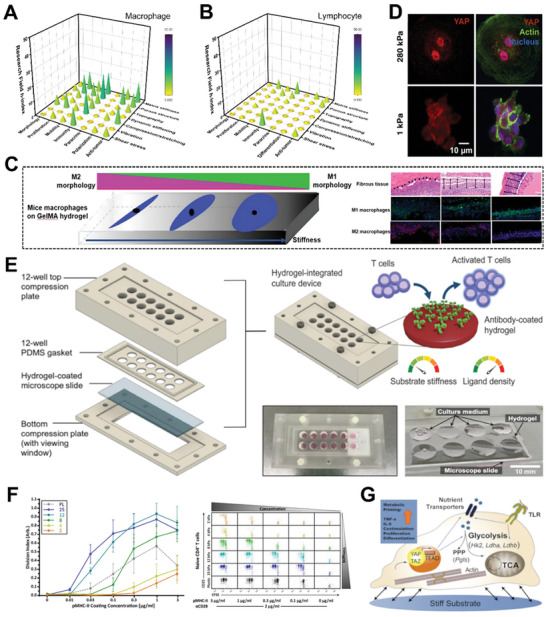
Mechanical stimulation on immune cell behaviors by hydrogel‐based platforms. 3D bar graphs of mechanical stimulation on A) macrophage and B) lymphocyte for various cell activities and biomedical applications. The research conditions are evaluated based on h‐index of each field calculated by paper publication and citation data from *Scopus*. C) Macrophages seeded on stiff gelatin‐based hydrogels (GelMA) tend to M1 phenotype with increased spreading, while more macrophage infiltration but thinner fibrotic capsule formation is observed on soft surfaces. Reproduced with permission.^[^ ^269^ ^]^ Copyright 2020, American Chemical Society. D) Immunofluorescence confocal images of YAP clusters in macrophages cultured on gels. YAP nuclear localization elevates with the increase of gel stiffness.^[^ ^270^ ^]^ Copyright 2020, American Association for the Advancement of Science. E) Schematic illustration and images of hydrogel‐integrated culture device for T cell activation by tuning matrix stiffness and ligand density. Adapted with permission.^[^ ^279^ ^]^ Copyright 2020, American Chemical Society. F) Average division index and representative plots of CD25 expression of CD4^+^ T cell. Increased gel stiffness promotes CD4^+^ T cell proliferation and reduces the threshold dose of the required ligand. (Data are shown as means ± SEM, *n* ≥ 3.) Reproduced with permission.^[^ ^262^ ^]^ Copyright 2020, eLife Sciences Publications Ltd. G) Schematic illustration that substrate stiffness impacts dendritic cell metabolism and function through the Hippo‐signaling factor (TAZ) and ion channel (Piezo1). Reproduced with permission.^[^ ^282^ ^]^ Copyright 2020, Elsevier.

Immune cells, as the building blocks of the body's immune system, defend our body from all types of pathogens. Immune cells are found throughout the body and are concentrated in lymphoid organs. Generally, they are divided into three types: lymphocytes (i.e., T cells, B cells, and natural killer cells), neutrophils, and monocytes/macrophages.^[^ ^18^ ^]^ Given the central roles of immune cells in the immune system, immense efforts have been made over the past decade to delineate immune cell biology and how to manipulate their behaviors.^[^ ^261^ ^]^ As of December 2022, there are ≈12 000 registered clinical trials worldwide listed on ClinicalTrials.gov that use T cells for immunotherapy, especially in cancer treatment. Chimeric antigen receptor T cell (CAR‐T) therapy has sparked substantial excitement in recent years, and since 2017, six CAR‐T products have been FDA‐approved and are already being used to treat leukemia, and, more recently, multiple myeloma. Researchers have discovered a number of biochemical entities such as signaling proteins (i.e., cytokines) and antibodies that regulate immune cell behaviors via molecular interaction and enzyme activity using biochemical and molecular biological tools. However, the impact of different mechanical factors on immune cell behaviors has been largely overlooked (**Figure** **7**A,B). One prominent reason for this omission is to the difficulty of applying biophysical stimuli to immune cells, which are often non/weak‐adherent, microscopic, and fragile.^[^ ^43^ ^]^ Nonetheless, researchers can now study the mechanical properties of immune cells in greater depth thanks to recent technological advances such as characterization techniques and cell culture platforms.

#### 4.4.1 Stiffness

Physical stiffness is an important mechanical stimulus that influences immune cell behaviors. This is because changes in matrix stiffness, such as those found in lymph nodes, are a natural process that serves as guidance for immune functions.^[^ ^18^ ^]^ In general, matrix/cell stiffness acts as a costimulatory signal for immune priming and provides the ideal condition for immune activation.^[^ ^262^ ^]^ Most immune cells respond to stiffness changes in their surrounding through alteration in proliferation, migration, phenotypic shift, and immune activation. Furthermore, tissue or matrix stiffness is a common pathological marker for immune organs and is associated with disease pathogenesis and progression. To that end, Singh's group designed bioartificial hydrogels (lymphoma cells were seeded and expanded in the hydrogel to form organoid) that can simulate the stiffness of healthy and neoplastic lymph nodes. As a result, they discovered that lymphoid tissue stiffness affected the progression, drug response, and B‐cell receptor signaling of diffused large B‐cell lymphoma (malignant B cell tumor) in a molecular subtype‐dependent manner.^[^ ^263^ ^]^ Researchers frequently overlook the stiffness‐dependent immune cellular response, which necessitates further investigation to determine the underlying mechanisms and therapeutic potentials.

Macrophages are a heterogeneous population of immune cells that serves as the primary effector of inflammatory response to injury or infection.^[^ ^264^ ^]^ They are found as resident cells in tissues, where they are not only patrol the surroundings and remove pathogens/apoptotic cells, but also induce inflammation and activate the immune system.^[^ ^265^, ^266^ ^]^ When exposed to various microenvironmental stimuli, macrophages become activated and exhibit phenotypic polarization, which can be broadly categorized into two extremes: classically activated (M1‐like, proinflammatory type) and alternatively activated (M2‐like, anti‐inflammatory type).^[^ ^261^, ^267^, ^268^ ^]^ Recently, a few hydrogel‐based studies demonstrated that macrophages are mechanosensitive, and that mechanical stiffness can influence their phenotypes. For example, it has been proven that high matrix stiffness (i.e., hydrogel modulus) activates macrophages into proinflammatory (M1) phenotype. Specifically, Zhuang et al. discovered that a stiffer matrix induced macrophage spreading and M1 phenotypic polarization, which is characterized by increased proinflammatory cytokine secretion, when compared to the macrophages cultured in softer hydrogel (Figure 7C).^[^ ^269^ ^]^ The author inferred that the gel stiffness promoted cell focal adhesion and F‐actin microfilaments formation, which resulted in macrophage activation and phenotype shift via the mechanotransduction pathway. Another possibility is that a stiff matrix promotes YAP nuclear localization. According to Meli et al., increasing the matrix stiffness allowed more YAP to enter the nucleus of macrophages, thus enhancing the cell's responsiveness to inflammatory signals (i.e., activated status) such as lipopolysaccharide (Figure 7D).^[^ ^270^ ^]^ The discovery of stiffness‐induced macrophage polarization suggests a promising strategy for controlling inflammation processes. In future, stiffness‐tunable hydrogels may have great clinical potential in inflammation/host response‐controllable wound dressing and implant.^[^ ^271^, ^272^ ^]^ Furthermore, matrix stiffness can also serve as a supporting role in biochemical stimulation. It was found that rigid matrix enhances the cell polarization induced by biomolecular signals (e.g., lipopolysaccharide^[^ ^271^ ^]^) and cytokines/chemokines (e.g., IL‐4^[^ ^273^ ^]^). On the other hand, a small number of experimental results validated that neighboring cells residing around macrophages can influence their cell responses. According to Coburn et al., when macrophages were cocultured with fibroblasts in a stiff hydrogel, the presence of fibroblasts promoted anti‐inflammatory activity (e.g., increased levels of IL‐10), while the absence of fibroblasts increased proinflammatory activity.^[^ ^274^ ^]^ This result suggested that, when compared to the effect of matrix stiffness, fibroblast played a more dominant role in regulating the inflammatory activity of macrophages.

Lymphocytes are another major subtype of immune cells that circulate in the blood and concentrates in lymphatic organs such as lymph nodes. In adults, ≈20–40% of leukocytes (white blood cells) are lymphocytes, which are the major components of the adaptive immune system that develops specific responses to early antigenic stimuli.^[^ ^18^ ^]^ T lymphocytes (or T cells) have been shown to sense and respond to mechanical stimuli through their surface receptors and cytoskeleton. T cell receptors (TCRs), such as the TCR–CD3 complex, function as mechanosensors in addition to recognizing antigen fragments and activating the immune system.^[^ ^275^ ^]^ In relation to that, external mechanical cues are considered as a crucial regulator for T‐cell immune functionality because they not only modulate T‐cell activities like deformation, migration, and infiltration, but also mediates T‐cell recognition and signaling.^[^ ^275^, ^276^ ^]^ Matrix stiffness is regarded as one of the most important mechanical factors regulating T‐cell behaviors during immune response. To study how matrix rigidity influences T‐cell activation during the antigen‐presenting process, T‐cells are usually seeded on the gel with a specific elastic modulus. Judokusumo et al. seeded mouse Naive CD4^+^ T cells on ligand‐presenting (anti‐CD3/CD28) PAA gels, and they found that IL‐2 secretion was higher in rigid gel (≥25 kPa) than in softer gel (10 kPa), suggesting that rigid matrix could enhance T cell activation.^[^ ^277^ ^]^ Moreover, Hickey et al. cultured CD8^+^ T cells on a stiffness‐modulable matrix composed of hyaluronic acid and stimulatory biomolecules.^[^ ^278^ ^]^ By tuning the hydrogel elastic modulus, this platform was able to control T cell signaling and phenotype, and it evidently expanded rare antigen‐specific T cells to a larger number, which is significant for using adoptive cell transfer to fight cancer. In addition, this work validated the role of hyaluronic acid as a T cell stimulating matrix by introducing additional signaling component that interacts with T cells and influences early cellular behaviors (e.g., priming, proliferation, activation). However, CD8^+^ T cells expanded better on soft hydrogels (0.5 kPa) than stiff ones (≥1 kPa), contradicting previous research that found a link between matrix stiffness and T cell activation. Chin et al. found similar result with Jurkat T cells showing higher activation (i.e., higher IL‐2 secretion level) and lower proliferation on stiffer matrix (Figure 7E).^[^ ^279^ ^]^ The observation demonstrates that, besides biochemical approaches, matrix stiffness can be used to control the balance between T cell stimulation strength and proliferative capacity. Soft matrices, for instance, are advantageous for achieving a comparable poststimulation proliferation rate while avoiding T cell exhaustion. Furthermore, hydrogel is being used to investigate the impact of mechanical properties of antigen‐presenting cells (APCs) such as dendritic cells (DCs) on T cell functions. Hypothetically, the change in DC cortex stiffness appears to alter T cell–DC interaction during T cell priming. Blumenthal et al. used a PAA hydrogel coated with stimulatory ligands to mimic DC cortex and discovered that a rigid surface reduced the threshold required for T cell activation in comparison to softer hydrogels (i.e., immature DCs) (Figure 7F).^[^ ^262^ ^]^ The result elucidates that DC cortical stiffness acts as a costimulatory signal for T cell priming, most likely because a stiffer surface allows T cells to exert more forces through TCR–antibody interactions and induces TCR complex conformational changes, resulting in a more effective signaling.^[^ ^277^, ^280^, ^281^ ^]^ Furthermore, DC is a mechanosensitive cell that responds to changes in environmental stiffness. Chakraborty et al. reported that when the stiffness of gel elevates, the static tension primes DCs metabolism and promotes inflammatory function through the downstream Hippo‐signaling factor, TAZ, or calcium ion channels (e.g., piezo) even without stimulation of pattern recognition receptor (Figure 7G).^[^ ^282^ ^]^ Similar stiffness‐dependent immune activation was also observed in B cells.^[^ ^283^ ^]^ Recent findings on using matrix stiffness to regulate T cell expansion and immune functions, when combined with recent strategies on hydrogel stiffening/softening technology, shed light on the potential of stiffness‐varying hydrogel as T cell synthetic bioreactors. For example, patient‐derived T cells can be cultured in softened matrix to promote proliferation, and then the hydrogel can be stiffened to activate T cells for better immune function. This will be particularly useful in immunoengineering applications such as CAR‐T therapy, as patient‐derived immune cells are fragile and limited in number.

#### 4.4.2 Topography

In addition to physical stiffness, researchers have utilized several hydrogel‐based platforms to study the effects of other material mechanical properties on immune cell behaviors, such as pore size,^[^ ^284^ ^]^ surface topography,^[^ ^285^ ^]^ and internal architecture.^[^ ^286^, ^287^, ^288^ ^]^ Nevertheless, the majority of existing work was conducted on 2D or 2.5D hydrogel systems. Although force‐induced immune cell activities were observed, these experiments were difficult to represent the native 3D physiological condition in which the immune cells reside.^[^ ^6^, ^289^ ^]^ For example, in 3D tissue microenvironment, encapsulated cells experience mechanical cues from multiple dimensions, whereas in 2D substrate, applied forces only act on the cell–material contact surface. This motivates future studies into how matrix mechanical cues influence immune cell behaviors in 3D configurations. Moreover, few studies have been conducted to understand how dynamic mechanical stimuli (e.g., flow‐induced shear stress) influence immune cell behaviors in comparison to static mechanical stimuli.

#### 4.4.3 Flow‐Induced Shear Stress

Flow‐induced shear stress is a more significant biomechanical feature than matrix mechanical features when considering the circulation of immune cells in blood/lymphatic system. Recent research has demonstrated that stress exerted by blood and lymph shear flow affect a series of immune functions including immune cell homing, APC–lymphocyte communication, and lymphatic metastasis in lymphoid organs. Integrative model combining hydrogel and microfluidic platforms are commonly deployed to investigate the effects of flow‐induced shear stress.^[^ ^290^ ^]^ Encapsulated immune cells are subjected to dynamic mechanical stress from continuous fluid flow in a hydrogel‐based microfluidic system, and this strategy has been widely used to simulate flow conditions in the lung,^[^ ^291^ ^]^ kidneys,^[^ ^292^ ^]^ and vascular network.^[^ ^293^ ^]^ Nonetheless, most of the abovementioned works emphasized on one or two types of immune cell activations (under stiffness stimulation). To fully comprehend the complex interplay between multiple cell components and flow shear, other immune cell types or cell clusters must be cocultured in flow simulation experiments. For instance, to fabricate an artificial lymph node‐mimic model, we could culture T/B cells in a gel with predefined stiffness to mimic the T/B cell zone, and macrophages as well as DCs could be seeded in the surrounding chambers, which correspond to medullary and subcapsular sinus structures. Compared to other cell types, the study of mechanical application of immune cells is still in its infancy, and we believe that as more attention is paid to this, possibly motivated by the clinical use of immune cells, the field of immunoengineering will continue to grow.

### 4.5 Endothelial Cell

**Figure 8:**
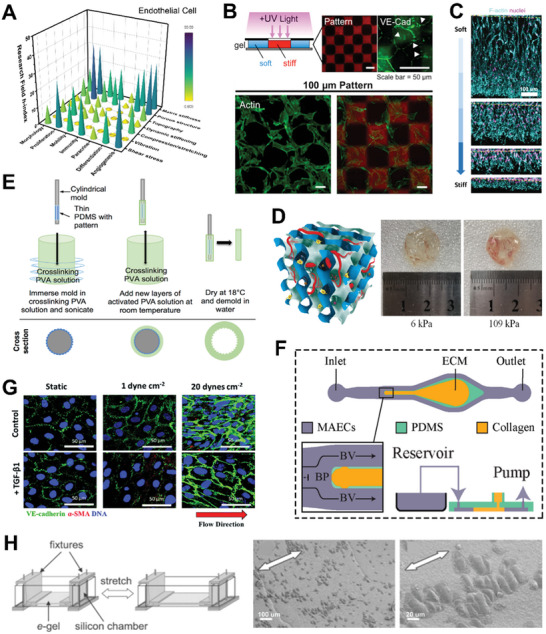
Mechanical stimulation on endothelial cell behaviors by hydrogel‐based platforms. A) 3D bar graph of mechanical stimulation on endothelial cells for various cell activities and biomedical applications. The research conditions are evaluated based on h‐index of each field calculated by paper publication and citation data from *Scopus*. B) Schematic illustration of photopatterned hydrogel with stiffness heterogeneity, soft (black) and stiff (red) regions. Confocal images of 100‐µm‐pattern hydrogel indicate that endothelial cells preferentially grow on stiff ones during monolayer formation, compared to soft matrix regions. Reproduced with permission.^[^ ^306^ ^]^ Copyright 2017, American Chemical Society. C) Composite fluorescence images that endothelial cells have enhanced angiogenesis sprout length on low‐stiffness hydrogel. Dashed yellow lines indicate cell channel position. Reproduced with permission.^[^ ^310^ ^]^ Copyright 2017, Springer Nature. D) Images show that stiff hydrogel matrix supports and promotes arterial‐venous differentiation of endothelial progenitor cells. Reproduced with permission.^[^ ^311^ ^]^ Copyright 2018, Wiley‐VCH. E) Schematic illustration of the dip‐casting approach to form luminal microsize patterns on tubular hydrogel. Reproduced with permission.^[^ ^313^ ^]^ Copyright 2016, Elsevier. F) Schematic illustration of gel/microfluidic device to learn bifurcating shear flow at the vessel branch point. Reproduced with permission.^[^ ^324^ ^]^ Copyright 2019, MDPI. G) Immunocytochemistry images elucidate that shear stress promotes cell transformation from endothelial to mesenchymal cell, with increased alpha‐smooth muscle actin (*α*‐SMA). Reproduced with permission.^[^ ^325^ ^]^ Copyright 2016, Royal Society of Chemistry. H) Schematic illustration of gel stretcher and images of scanning electron microscope that endothelial cells orient perpendicularly to the stretch direction. Reproduced with permission.^[^ ^339^ ^]^ Copyright 2008, Elsevier.

Endothelial cells form a continuous single‐cell‐layer wall known as the endothelium, which covers the inner surfaces of the circulatory systems including blood and lymphatic vessels. Apart from forming semipermeable barriers between vessel/tissues and restricting fluid movement, endothelial cells regulate blood flow, develop vascular network (e.g., vasculogenesis and angiogenesis), and participate in hemostatic processes by secreting a variety of proteins.^[^ ^294^, ^295^, ^296^, ^297^ ^]^ Given the similar characteristics and origins, endothelial cells are frequently considered as a specialized type of epithelial cells, as both of them serve as the interface between the internal and external environments. Nevertheless, endothelial and epithelial cells differ significantly in terms of morphology, location, and function.^[^ ^298^ ^]^ Endothelial cells are crucial in biomedical applications involving regenerative medicine, and researchers are currently attempting to engineer the cells to reconstitute certain functional circulatory systems and regulate their in vivo behaviors. As of December 2022, there are over 200 registered clinical trials using endothelial cells to investigate the therapeutic effect on vascular diseases listed on ClinicalTrials.gov. Several works have reported that endothelial cells are mechanosensitive and can be affected by several intertwined physical cues divided into two broad categories: mechanical features of matrix/substrates and fluid‐derived dynamic forces.^[^ ^111^, ^299^, ^300^, ^301^ ^]^ Matrix‐derived biophysical cues such as stiffness, porous structure, topography, and curvature can influence endothelial cells by exerting static stresses on the cell basal surface.^[^ ^302^, ^303^ ^]^ On the other hand, due to their constant exposure to blood/lymphatic flow, endothelial cells have the ability to sense and respond to fluid‐derived dynamic forces (**Figure** **8**A). Such dynamic forces include flow‐induced shear stress, compressive blood pressure, and circumferential/axial tensile strain.^[^ ^111^ ^]^ This segment will focus on how mechanical stimuli influence endothelial cell behaviors and functions. Other nonphysical factors affecting the endothelial cells function (e.g., biochemical cues) have been comprehensively reviewed elsewhere.^[^ ^304^, ^305^ ^]^

#### 4.5.1 Stiffness

Endothelial cells show stiffness preference in cell migration, moving preferentially from softer to stiffer region,^[^ ^306^ ^]^ and possess better migratory ability on the stiffer gel. In this regard, Lampi et al. discovered that matrix stiffness heterogeneity (i.e., soft–stiff patterned matrix) could rearrange cell–matrix and cell–cell contact based on the endothelial growth preference, thereby disrupting the integrity of the endothelial monolayer junction (Figure 8B).^[^ ^306^ ^]^ Nonetheless, in a similar experiment conducted on 3D hydrogel platform, the endothelial cell displayed a longer migration distance on the softer gel.^[^ ^307^, ^308^ ^]^ One potential reason is that when endothelial cells are encapsulated in 3D setting, the surrounding rigid matrix forms spatial impedance for both cell movement and cytoskeleton spreading.^[^ ^82^ ^]^ Additionally, the temporal hypoxic/nutrient gradients caused by gel thickness affect the endothelial cell behaviors, making this a feature to consider for vascular development in 3D ECM environment.^[^ ^309^ ^]^ Moreover, Trappmann et al. concluded that low hydrogel stiffness enhanced angiogenesis sprout length and caused leading cells to adopt an open‐branched morphology with longer filopodia (Figure 8C).^[^ ^310^ ^]^ On the other hand, Xue et al. successfully generated new blood vessels with visible tubular structures in dextran hydrogels and subsequently modulated the arterial‐venous differentiation of endothelial progenitor cells by varying the gel stiffness (Figure 8D).^[^ ^311^, ^312^ ^]^ It was discovered that stiffer matrix induced the endothelial progenitor cells to differentiate into arterial phenotypes, whereas softer matrix promoted differentiation into venous phenotypes. The soft and stiff matrix rigidities deployed in the experiment are consistent with the artery (≈50–150 kPa) and vein (≈3–50 kPa) found in vivo. The studies presented new protocols for manipulating vasculogenesis or angiogenesis by modulating ECM stiffness. While appropriate matrix stiffness yields encouraging results for tissue engineering utility, it is essential to note that changes in the matrix can also result in endothelial pathological condition or related diseases.

#### 4.5.2 Topography

Matrix topography is another important mechanical cue guiding endothelial cell behaviors. In vivo, the vascular endothelium tightly anchors to the vascular basement membrane, which is a continuous, thin layer (30–500 nm) with complex surface topography. This provides a multiscale isotropic or anisotropic topographical environment for endothelial cells.^[^ ^302^ ^]^ Poly(vinyl alcohol) (PVA) is a popular hydrogel material for creating various topographies. Planar PVA hydrogels could be patterned with micro‐/nanosized surface topographies such as grating, pillar, and convex–concave lens structure using casting and nanoimprint lithography, and the surface modifications could significantly improve endothelial cell density and adhesion in comparison to unmodified PVA hydrogel.^[^ ^313^ ^]^ In order to integrate topographical cues into 3D platforms, Yim's group developed a dip‐casting approach to achieve luminal microsize patterns on tubular PVA hydrogel, and their method could potentially be applied as a small diameter vascular graft. (Figure 8E).^[^ ^313^, ^314^, ^315^ ^]^ In a preliminary animal study, the 3D patterned vascular PVA graft (with a 2‐µm grating on the internal surface) exhibited improved patency and endothelialization after 20 days, whereas unmodified PVA graft was occluded in rat aorta.^[^ ^313^ ^]^ Besides, gelatin methacrylate (GelMA), a modified gelatin with an unsaturated group (i.e., C=C), was also utilized to fabricate topographically patterned hydrogel by UV crosslinking. For example, Rizwan et al. developed a micropillar GelMA hydrogel film with high mechanical strength. Subsequent experiments revealed that human corneal endothelial cells seeded on the patterned surface had tight cell–cell junctions, high cell density, and homogenous cell size.^[^ ^316^ ^]^ However, in comparison to PDMS substrate, it is still challenging to fabricate GelMA hydrogel with precise topographical patterning due to a lack of material mechanical strength and fabrication limitations. Aside from using macromolecules with better mechanical features, researchers have been able to achieve complex topographies on soft hydrogels using advanced technologies such as hydrogel laser carving/etching^[^ ^317^, ^318^, ^319^ ^]^ and volumetric additive manufacturing.^[^ ^320^, ^321^, ^322^, ^323^ ^]^ For more information on techniques for fabricating patterned hydrogels, readers are encouraged to read other published reviews.^[^ ^29^, ^303^ ^]^

#### 4.5.3 Flow‐Induced Shear Stress

Endothelial cells are subjected to fluid shear stress as a result of tangential friction between the endothelium basal surface and blood/lymphatic flow. A number of factors can disrupt the interaction between fluid flow and endothelial cells in this regard. The interactions between endothelial cells and fluid flow can be easily captured and observed by microscopy by integrating microfluidic technology with a hydrogel matrix that can provide a well‐controlled 2D/3D microenvironment.

Recent studies highlight the significance of flow stress to the endothelium in affecting angiogenesis. Akbari et al. investigated the effect of luminal flow at the vessel branching point and observed that bifurcating shear flow could hamper the formation of angiogenic sprouts (Figure 8F).^[^ ^324^ ^]^ Interestingly, the simultaneous application of transmural flow was shown to restore sprouting, suggesting that transmural flow has a competing effect against bifurcating flow and plays a regulatory role at vessel branching site. Furthermore, altering flow‐induced shear stress can also influence endothelial cell phenotype transformation. Mina et al. discovered that lower flow stress coupled with TGF‐*β*1 treatment promotes endothelial to mesenchymal cell transformation (increased *α*‐SMA level), which is followed by increased cell invasion and ECM remodeling (i.e., 3D collagen gel). By contrast, a higher magnitude of flow stress suppressed this process (Figure 8G).^[^ ^325^ ^]^ A plausible explanation for the observation is that slow flow results in wider cell–cell gap junctions, compromising endothelial monolayer integrity and leading to mesenchymal‐like characteristics. It is noteworthy that flow‐induced shear stress is impacted by other vessel matrix mechanical cues (e.g., stiffness, thickness, curvature), and the cooperative effects often result in abnormal physiological performance of endothelium, such as atherosclerosis and tumorigenesis.^[^ ^326^, ^327^, ^328^, ^329^ ^]^

Flow‐induced traction or intercellular stress is another crucial mechanical cue regulating endothelial cell behaviors in vivo. Perrault et al. observed that endothelial cells have an acute but reversible response to slow flow (similar to interstitial flow) due to the heightened traction and intercellular stress,^[^ ^330^ ^]^ which is also supported by Steward et al.^[^ ^331^ ^]^ As interstitial flow can induce the formation of new vessels in vivo,^[^ ^332^ ^]^ it is speculated that enhanced traction and cell–cell stress are required to initiate the process. In addition to flow‐induced traction force, hydrostatic pressure from fluid flow may disrupt the vascular endothelial–cadherin junction, leading to a multilayer endothelium structure with increased intercellular gap and permeability.^[^ ^333^, ^334^, ^335^ ^]^ This creates favorable conditions for endothelial cell migration and proliferation, which results in the formation of new vessels.

While microfluidic devices can precisely stimulate fluid flow in vitro, there are still hurdles to their widespread adoption in endothelium‐related research. In most microfluidic platforms, the absence of associated cells in the fluid flow (e.g., blood cells) causes a deviation in the observation. This is because the flowing cells can substantially alter the near‐wall flow field, thus influencing the shear force on the surface of endothelial cells according to the recent studies.^[^ ^336^, ^337^ ^]^ The actual blood and body field (a non‐Newtonian fluid) is more viscous and has shear‐thinning behavior when compared to the fluid flow of the experimental medium (which is commonly in the form of a Newtonian fluid).^[^ ^338^ ^]^

#### 4.5.4 Dynamic Stretching

Pulsatility, which is commonly caused by rhythmic heartbeat, is another vital property affecting endothelium, particularly on arteries (i.e., arterial endothelial cell). On some microfluidic devices, pulsatile flow with periodic directional reversal and oscillation has recently been simulated. Nonetheless, only a few studies have looked at the pulsatile effects on the matrix, such as vessel contractility and elastic retraction. Several studies have illustrated that the pulsatile behavior of matrix affects the endothelial cells, which is a scenario shared by epithelial cells. For example, Kanayama et al. demonstrated that the endothelial cells oriented perpendicularly to the stretch direction by stretching cell–seeded gel on a uniaxial stretcher (Figure 8H).^[^ ^339^ ^]^ In addition, while recent studies about individual matrix (i.e., static) or flow‐derived (i.e., dynamic) mechanical stimuli on endothelium have greatly broadened our understanding of cell mechanobiology, endothelial cells, in fact, experience a complex integration of all mechanical cues in vivo. These mechanical cues are often interdependent. Aside from modulating a single cellular process, multiple mechanical stimuli may have synergistic or antagonistic effects, or one may modulate the effect of other cues and change cell behaviors. It is hence important to study pathogenesis in relation to a set of environmental cues and how this interplay leads to the disease condition. Therefore, the further development of hydrogel/microchannel platforms that integrate multiple mechanical cues is required to meet the evolving experimental requirement in pursuit of more refined and targeted research.^[^ ^111^ ^]^

## 5 Mechano‐Stimulated Hydrogel to Enhance Clinical Translation

Based on the literature reviewed in Section 4, we found that each cell type has its “preferred” mechanical stimulation factors. Considering the diversity of cell types, mechanotransduction pathways, and native microenvironment, different static/dynamic mechanical stimulations can have dramatically different effects on cells (**Table** **1**). For example, in our body, skin‐derived cells such as fibroblast and epithelial cells generally experience continual stretching and compression from body activities, while endothelial cells experience strong shear stress from blood flow. In addition, matrix mechanical features (i.e., static mechanical stimulation) have obvious impacts on adherent cells with spreading morphology, while generating weaker effects on suspension cells. In Table 1, we indicate the level of evidence supporting the impact of each type of mechanical stimuli on different cell types. These results highlight the importance of using targeted mechanostimulation and the significance of mechanical cocktails in cellular bioengineering.

**Table 1:** The impact of mechanical stimulation on different cell types. The level is estimated based on the effect of each mechanostimulation in literatures, research field h‐index, and cell growth mechanical environment

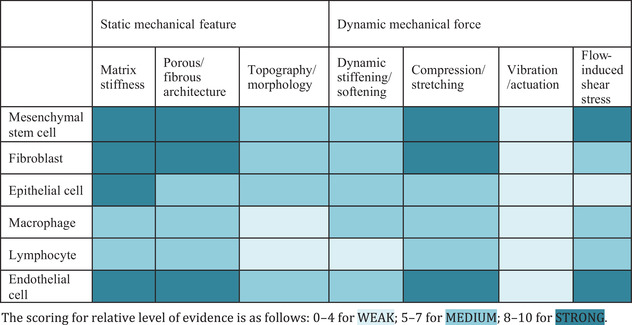

The final score is based on “publication number + citation condition + mechanostimulation.” For publication number (data from *Scopus*): 3 points for ≥300; 2 points for ≥100; 1 point for ≥40; 0 point for <40. For citation number (data from *Scopus*): 3 points for ≥10 000; 2 points for ≥1000; 1 point for ≥100; 0 point for <10. For mechanostimulation effect (average significant difference level in highest cited 20 papers): 3 points for <0.001; 2 points for <0.01; 1 point for <0.05; 0 point for no significant. There is also an additional point for confirming that one mechanical feature can affect multiple cellular biofunctions.

**Figure 9:**
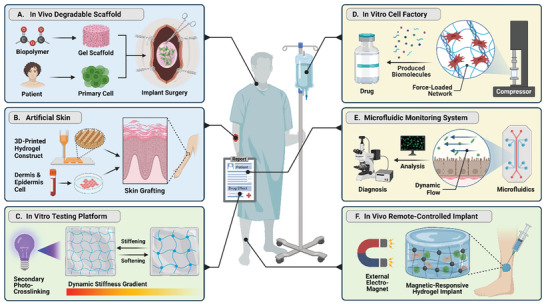
Schematic illustration of hydrogel biomedical applications based on static and dynamic mechanical factors, including A) in vivo degradable scaffold with programmable stiffness and microstructure, B) 3D‐printed artificial skin with specific surface topography, C) dynamic stiffening/softening platform for patho‐biological study, D) dynamic mechano‐induced in vitro cell factory producing biomolecules, E) gel/microfluidic device with dynamic flow for drug screening and mechanistic insight, and F) magnetoresponsive in vivo hydrogel implant.

With rapid progress in biopolymer synthesis and biomaterial fabrication techniques, it is now possible to mass‐produce biocompatible hydrogel scaffolds with precise material mechanical features such as stiffness, internal microstructures, and topography. The availability of microcircuitry and scalable device populations enables the application of specific dynamic mechanical forces in clinical settings, achieving effects that static stimuli cannot provide (**Table** **2**). With this, cell–biomaterial composites providing targeted mechanical stimulations hold great promise for use in clinical translations including biodegradable implants, in vivo/vitro screening platforms, cell manufacturing, and mechanomedicine (**Figure** **9**).

**Table 2:** The stimulation amplitude/parameter of dynamic mechanical stimulation

Cell type	Dynamic mechanical stimuli	Mechanical stimulation amplitude/parameter	Induced cell behaviors	Refs.
Fibroblast	Dynamic stress	3‐day cyclic loading, 0.1 Hz	Enhanced proliferation, skin substitute production	[227]
	Matrix stretching	Cyclic equibiaxial stretching (7%, 15%, or 20% at 1 Hz)	Increased collagen production, *α*‐SMA expression	[231]
		2–16% cyclic stretch, 6 or 24 h day^−1^, 8 days	Increased collagen density	[228]
	Compression	5, 15, 25, 35 g cm^−2^, 24 h	Max proliferation at 25 g cm^−2^	[340]
Epithelial cell	Stretching	20% strain, 45 min, 0.5 Hz	Cell reorientation (transverse to stretching direction)	[258]
		10%‐strain stretching	Epithelial crack and healing	[259]
		0–15% cyclic strain, 0.2 Hz	Increased cell–cell interaction	[260]
	Compression	Loaded with 150 g weights	Improve skin reconstitution	[341]
Endothelial cell	Flow‐induced shear stress	>10 dyn cm^−2^	Sprouting, matrix invasion	[342]
		20 dynes cm^−2^ steady shear	Reduced endothelial‐to‐mesenchymal transition	[325]
		1.2 Pa laminar fluid shear	Retarded elongation, alignment	[330]
Mesenchymal stem cell	Compression	10% peak compressive sinusoidal strain, 1 Hz, 5% compressive tare strain	Enhanced chondrogenic differentiation, survival	[196]
		0–2.5% strain, 1 Hz, 1‐h ON +23‐h OFF	Distinct collagen expression, mineral deposits	[197]
		10 kPa, 0.25 Hz, 1 h day^−1^, start day 1 or 21	Regulated chondrocyte markers, hypertrophy	[199]
	Stretching	±1%/2 days, 4–16%, 50% ON/OFF (3, 6, or 9 h)	Optimal regime for MSC matrix production	[190]
	Vibration	0.3, 3, or 6 g loads, 100 Hz	Enhanced osteogenesis (low/medium), inhibited (high)	[173]
	Force loading	1.6 pN per microparticle, 0.1 Hz	Increased MSC behaviors, osteogenesis, reduced chondrogenesis	[153]

For example, by leveraging cutting‐edge 3D‐printing and bioimaging techniques, topographical patterns can be replicated in hydrogel scaffolds with nanometer precision in a structurally defined and controllable manner (e.g., gradient). This is useful in fabricating tissue with specific structures such as artificial skin with fibroblasts and epithelial cells (e.g., keratinocyte), or organs with different function microzones such as lymph node (i.e., T/B cell zone), which are directed by matrix stiffness and porous structure.^[^ ^18^, ^261^ ^]^ These artifacts in most cases are constructed by degradable bio‐ink which will be gradually disintegrated and replaced by regenerated tissues and ECM components.^[^ ^343^ ^]^

In addition to tissue engineering, by using secondary crosslinking (e.g., photochemistry and enzyme) or external stimuli (e.g., magnetic field and temperature), it is possible to modulate stiffening/softening process on specially crafted hydrogels,^[^ ^26^ ^]^ providing researchers with a reliable drug screening platform to simulate the drug/therapeutic efficacy under stiffening/softening conditions. This is particularly promising in tumor therapy because when a tumor forms, cancer cells interact reciprocally with the stromal cells and external matrix through both mechanical and biochemical means to progressively remodel the environment (e.g., stiffening the surrounding ECM), making it more conducive for metastasis and invasion.^[^ ^344^ ^]^ Previous studies have discovered that matrix stiffness has a strong influence on chemotherapeutic responses of cancer cells to doxorubicin (chemotherapy medication),^[^ ^345^, ^346^ ^]^ and others demonstrated that decreased matrix modulus could induce downregulation of cell migration^[^ ^347^ ^]^ and angiogenesis outgrowth,^[^ ^140^ ^]^ loss of detoxification capacity,^[^ ^348^ ^]^ and enhanced T cell infiltration.^[^ ^349^, ^350^ ^]^ Collectively, these findings highlight that matrix dynamic mechanical cues are crucial in the interplay between tumor‐associated cells and the microenvironment within the distinct, heterogeneous tumor site.

For dynamic mechanical stimulation, a common method to exert dynamic stress or generate gel deformation is through external devices such as mechanical compressors or stretchers. As dynamic mechanical stimulation can boost cellular paracrine activities,^[^ ^165^, ^351^, ^352^ ^]^ it is possible to establish a mechano‐accelerated hydrogel platform acting as an in vitro cell factory to promote biomolecule production of exosomes, cytokines, and growth factors. Taking advantage of a flexible “ON/OFF” system and tunability of external dynamic stimulation, cellular paracrine activities can be paused accordingly to avoid cell exhaustion and adaption and excessive activation. Besides, a hydrogel‐based microfluidic system can exert dynamic flow‐induced shear stress on cells offering a powerful in vitro real‐time monitoring platform for drug tests and mechanistic observation.^[^ ^18^ ^]^ Moreover, as dynamic force is capable of directing cell orientation, it is viable to generate specific cell alignment for tissue regeneration purposes, including potential artificial skin graft.^[^ ^227^ ^]^ Another potential application for these dynamic hydrogel systems is for cell mechanical preconditioning, especially for cell manufacturing. Recently, it has been found that mechanical preconditioning is essential to maintain primary cell functions and withstand static/dynamic loading expected in vivo, particularly, for stem cells.^[^ ^36^, ^353^ ^]^ FDA guidance specifically calls for the inclusion of mechanical testing of cell products designed for applications such as cartilage and bone regeneration which are ≈18% of existing clinical trials using mesenchymal stem cells.^[^ ^354^ ^]^

## 6 Conclusions and Outlook

The field of cell mechanostimulation is garnering growing interest in the scientific and industrial community for its capability to unravel the intricacy of cell mechanobiology and its promising outlook in regenerative medicine, immunoengineering, cell manufacturing, and mechanomedicine. Hydrogels featuring static and dynamic mechanical properties are poised to be one of the enablers of future studies. Static mechanical features, particularly biophysical properties, of hydrogel are still the gold standard in designing and building scaffolds for different tissue engineering use cases. Currently, the majority of mechanoactivation studies still fall under the scope of substrate/matrix stiffness and have already been applied clinically such as for cartilage/bone regeneration. However, it is important to acknowledge that for most current platforms, stiffness is mostly tuned by mass concentration and crosslinking rate of polymer. As such, varying stiffness will likely lead to resultant changes in other mechanical features of the matrix. Thus, the secondary effects of manipulating matrix rigidity should be given due consideration by researchers to avoid introducing confounding in experimental data interpretation.

It should be highlighted that, arguably, dynamic factor is a better choice than static parameter for biomechanical stimulations. In our body, cells in their native environments experiment dynamic environment across space and time (e.g., ECM remodeling and stiffness variation) as well as force magnitude and frequency (e.g., dynamic stress and shear force). It is thus critical to be as physiologically relevant as possible to study the basic biology process to strengthen translation. Also, it has been previously shown that the effects of static matrix stiffness or constant mechanical stimulation gradually vanishes because of cell adaptation.^[^ ^188^, ^189^ ^]^ The application of dynamic loading is believed to be able to alleviate the effects of cell adaptation by resetting cell mechanosensitivity and improving cellular mechanoresponses.^[^ ^190^, ^191^, ^192^ ^]^ In addition, considering the features of reversibility and flexibility, dynamic stimuli is more promising for future clinical application due to great controllability. One such application is to better balance different cell functions (e.g., proliferation and stemness of mesenchymal stem cells) to achieve optimal quality and quantity.^[^ ^153^ ^]^

Currently, with development in technologies such as 3D printing fabrication, functionalized biomaterials, microfluidic platform, and external actuation devices, dynamic mechanical stimulation is becoming more accessible for research and clinical translation. With a deeper understanding of how dynamic forces affect cell activities, the positive effects accompanying such mechanical stimulation can be exploited in biomedical applications. Several studies have revealed that certain cellular responses are uniquely observed only under dynamic stimulation and not in comparable static hydrogel studies.^[^ ^251^ ^]^ More than often, dynamic mechanical stimulation can be described as the variation of corresponding static stimulation with time. From a calculus perspective, dynamic mechanical force can be interpreted as a constantly changing static stress derived from matrix mechanical features, for example, the effect of dynamic compression can be seen as the cyclic changing stiffness (or stiffening) and such force variation elicits stronger cellular responses and longer stimulation effects.^[^ ^23^, ^24^ ^]^

Static mechanical stimulation or material biophysical feature remains the primary focus area of hydrogel technology for cell engineering. However, we firmly believe that with a deeper understanding of cell mechanotransduction and advanced biomaterial approach, dynamic mechanical stimuli would receive more attention and become clinically useful. One big challenge of deploying external devices for dynamic mechanical stimulations in clinical setting is that nontargeted tissues may be affected. Some new strategies such as localized ultrasonic vibration and magnetoactivation are currently being developed to exploit their noninvasive feature and remote‐control ability.^[^ ^252^ ^]^ Compared to conventional approaches using mechanical stretcher, these contactless stimulations are convenient and easy to use with fewer side effects such as skin irritation and surrounding tissue damage. However, more studies are required to eliminate long‐term side‐effects as both external ultrasonic vibration and magnetic field are known to elicit adverse effects on the human body.^[^ ^355^ ^]^ In comparison to static mechanical features, the experimental conditions with dynamic mechanical stimulations are more complex (e.g., force magnitude, frequency, duration, etc.) and data interpretation is more challenging. This problem might be circumvented with machine learning and AI‐based approaches to screen for optimal conditions more efficiently.^[^ ^190^ ^]^ Through integrating innovations in hydrogel engineering, dynamic stimulation devices, and machine learning approaches, we believe that the research community can identify the best mechanical cocktail conditions to better mimic the physiological in vivo environment and exploit this concept for mechanocell reprogramming, manufacturing, and therapy.

## Conflict of Interest

The authors declare no conflict of interest.

## Author Contributions

Y.S. and X.Y.T. contributed equally to this work. Y.S. and A.T. conceptualized the idea of the review. Y.S. and X.Y.T. wrote initial drafts of the manuscript. K.Z.W. contributed to Section 4 writing and manuscript improvement. B.B., K.Z.W., A.R.K.K., J.L., and Z.L. contributed to data collection, graph making, and manuscript improvement. All authors contributed to writing and reviewing the manuscript and gave approval to the final version of the manuscript.
